# PharmVIP: A Web-Based Tool for Pharmacogenomic Variant Analysis and Interpretation

**DOI:** 10.3390/jpm11111230

**Published:** 2021-11-19

**Authors:** Jittima Piriyapongsa, Chanathip Sukritha, Pavita Kaewprommal, Chalermpong Intarat, Kwankom Triparn, Krittin Phornsiricharoenphant, Chadapohn Chaosrikul, Philip J. Shaw, Wasun Chantratita, Surakameth Mahasirimongkol, Sissades Tongsima

**Affiliations:** 1 National Biobank of Thailand, National Science and Technology Development Agency, Klong Luang, Pathum Thani 12120, Thailand; chanathip.suk@ncr.nstda.or.th (C.S.); pavita.kae@nstda.or.th (P.K.); chalermpong.int@nstda.or.th (C.I.); kwankom.tra@ncr.nstda.or.th (K.T.); oatkrittin@gmail.com (K.P.); chadapohn.chaosrikul@gmail.com (C.C.); sissades.ton@nstda.or.th (S.T.); 2 National Center for Genetic Engineering and Biotechnology, National Science and Technology Development Agency, Klong Luang, Pathum Thani 12120, Thailand; philip@biotec.or.th; 3 Center for Medical Genomics, Faculty of Medicine Ramathibodi Hospital, Mahidol University, Phayathai, Bangkok 10400, Thailand; wasun.cha@mahidol.ac.th; 4 Division of Genomic Medicine and Innovation Support, Department of Medical Sciences, Ministry of Public Health, Nonthaburi 11000, Thailand; surakameth.m@dmsc.mail.go.th

**Keywords:** pharmacogenomics, variant analysis, web-based-tool, next-generation sequencing, allele prediction, CPIC dosing recommendation, HLA, adverse drug reaction, variant effect prediction, bioinformatics

## Abstract

The increasing availability of next generation sequencing (NGS) for personal genomics could promote pharmacogenomics (PGx) discovery and application. However, current tools for analysis and interpretation of pharmacogenomic variants from NGS data are inadequate, as none offer comprehensive analytic functions in a simple, web-based platform. In addition, no tools exist to analyze human leukocyte antigen (HLA) genes for determining potential risks of immune-mediated adverse drug reaction (IM-ADR). We describe PharmVIP, a web-based PGx tool, for one-stop comprehensive analysis and interpretation of genome-wide variants obtained from NGS platforms. PharmVIP comprises three main interpretation modules covering analyses of pharmacogenes involved in pharmacokinetics, pharmacodynamics and IM-ADR. The Guideline module provides Clinical Pharmacogenetics Implementation Consortium (CPIC) drug guideline recommendations based on the translation of genotypic data in genes having guidelines. The HLA module reports HLA genotypes, potential adverse drug reactions, and the relevant drug guidelines. The Pharmacogenes module is employed for prioritizing variants according to variant effect on gene function. Detailed, customizable reports are provided as exportable files and as an interactive web version. PharmVIP is a new integrated NGS workflow for the PGx community to facilitate discovery and clinical application.

## 1. Introduction

Pharmacogenomics (PGx) is the study of how genetic variants contribute to phenotypic differences in drug response among individuals. PGx is used for personalizing drug selection to prevent adverse effects and to optimize therapeutic efficacy [1,2,3]. Although drugs are generally effective and used with low risks of adverse events, response to drugs varies among individuals. Knowledge of variants in genes associated with drug response (pharmacogenes) is vital for optimizing an individual’s therapeutic outcome [4]. Pharmacogenes include genes governing pharmacokinetics (PK), i.e., Cytochrome P450 genes, receptor/transporter genes (drug targets) governing pharmacodynamics (PD), and genes with functions related to immune-mediated adverse drug reactions (IM-ADR), i.e., human leukocyte antigen (HLA) genes.

The single nucleotide polymorphism (SNP) array is a genotyping technology that is popularly used to determine variants in pharmacogenes because it offers a fast and straightforward interpretation [5]. However, rare SNPs and some structural variants may not be detected using SNP arrays. Next-generation sequencing (NGS) is a promising alternative genotyping technology for PGx, and represents a reliable and effective tool to uncover both common and rare variants in pharmacogenes [6,7,8]. In the past ten years, NGS data from population genomic projects have been explored to understand population-specific genetic diversity in pharmacogenes [9,10]. Despite its potential, NGS has not yet been applied in routine clinical PGx [11,12] because there are only a few genes that show sufficiently strong genotype-phenotype association for clinical application. Moreover, the greater complexity of NGS data makes data processing challenging.

In addition to the rapid growth of genotyping technologies, several reference PGx-resources have been established to facilitate research and clinical implementation in this field. The Pharmacogenomics Knowledgebase (PharmGKB) is a PGx reference database of well-curated, clinically relevant data including actionable gene–drug associations, genotype–phenotype relationships and drug dosing guidelines [13]. The establishment of the Clinical Pharmacogenetics Implementation Consortium (CPIC) [14], from the collaboration between the Pharmacogenomics Research Network (PGRN) and PharmGKB, aims to expedite the efficient translation of genetic test results into actionable prescribing decisions by providing standard drug dosing guidelines for gene-drug associations that have strong supporting evidence. PharmVar [15] aims to standardize the nomenclature of variants found in pharmacogenes. PharmVar hosts expert-curated gene panels as well as other important pharmacogenes. The Drugbank project [16] provides comprehensive molecular information of drugs offered via an online web-based database, which also houses variants associated with drug responses. Despite the advancement of genotyping technologies and PGx knowledge resources, there is a need for a tool that comprehensively integrates analysis of pharmacogenomic variants from NGS data and PGx knowledge resources to facilitate PGx research and clinical application.

Several online PGx tools have been developed to predict drug responses from genotypic profiles that fulfill some, but not all requirements. Virtual Pharmacist [17] is a web-based tool that accepts input genotypic profiles from SNP array data or Variant Call Format (VCF) files obtained from NGS data. Virtual Pharmacist also accepts raw NGS data in FASTQ format and generates genotypic profiles for analysis. The tool matches genotypes with PGx reference databases and reports the variant-associated drug responses, along with guidelines for drug dosing, drug toxicity and treatment efficacy. The key reference PGx databases used by Virtual Pharmacist include the PharmGKB database and the DrugBank database. However, this tool does not provide users the information of the haplotype-based drug dosing recommendations. The electronic Pharmacogenomics Assistant (ePGA) [18] is another web-based PGx tool. It interprets genotypic data both at the single variant and haplotype levels and reports pharmacogenomic associations and drug dosing guidelines. However, the interpretations from ePGA are limited to information available in the PharmGKB database. At the time of writing, ePGA is no longer supported and is not functional. The most recent free PGx tool is PharmCAT [19], developed in collaboration between PGRN and PharmGKB. PharmCAT identifies the possible diplotype(s) of each pharmacogene, interprets drug-response phenotypes and outputs the associated CPIC drug dosing guidelines. PharmCAT was designed for clinical implementation and it focuses on the 16 pharmacogenes with CPIC drug dosing guidelines. Some pharmacologically relevant genes are not available in the current version, including the HLA gene family. Moreover, PharmCAT is a command line-operated tool that could be difficult to deploy in clinical settings, especially for bioinformatically inexperienced users. The g-Nomic tool [20] is a commercial implementation of PGx interpretation software designed to be used in clinical practice. However, g-Nomic was not designed for large-scale pharmacogenomic data analysis from SNP array or NGS data. PharmaKU [21] is a recently developed web-based tool that focuses on the translation of a person’s whole genome variant data (VCF format) into clinical recommendations. Utilizing the information from the PharmGKB database, the current version of PharmaKU provides CPIC guideline recommendations of 37 drugs based on diplotype inference in nine pharmacogenes using the Stargazer tool [22].

The currently available PGx tools offer services to avoid adverse drug reactions (ADRs) and inefficient drug use. The pharmacogenes interrogated by these tools concern mainly type A ADRs derived from the direct effects of drugs. Type A ADRs are preventable by dose adjustment or by using alternative drugs [23,24]. Type B ADRs, however, stem from either off-target receptors or drug allergies mediated by the immune system, and hence are also called immune-mediated ADRs or IM-ADRs. Although less common (representing about 20% of all ADRs), IM-ADRs pose a much higher burden as the underlying drug responses can be more severe. Variants in human leukocyte antigen (HLA) genes have been associated with IM-ADRs [25], and some actionable HLA gene–drug interaction guidelines have been issued, including HLA-B*57:01 associated with hypersensitivity to abacavir, HLA-B*15:02 associated with carbamazepine-induced Steven-Johnson syndrome (SJS)/toxic epidermal necrolysis (TEN), and HLA-B*58:01 associated with allopurinol-induced SJS/TEN [26]. Accurate HLA genotyping is challenging because of hyperpolymorphism and reference divergence [27,28]. Nevertheless, several computational methods have been developed for HLA genotyping from NGS data, which can provide up to 99% accuracy [28,29,30]. Despite the availability of such methods, HLA genotyping has not yet been incorporated in any of the aforementioned PGx tools.

We developed a new pharmacogenomic software called “PharmVIP” (Pharmacogenomic Variant Analysis and Interpretation Platform) as a one-stop web-based tool, offering the Guideline, HLA, and Pharmacogenes modules for analysis and interpretation of genomic variants in all pharmacogenes, including HLA genes not analyzed by any other PGx tool. We envision that it will be well suited for exploratory research and discovery of new pharmacogenetics factors. As a web-based tool, the software is easy to use, even by researchers unfamiliar with NGS data analysis or command-line tools.

## 2. Results and Discussion

**Figure 1:**
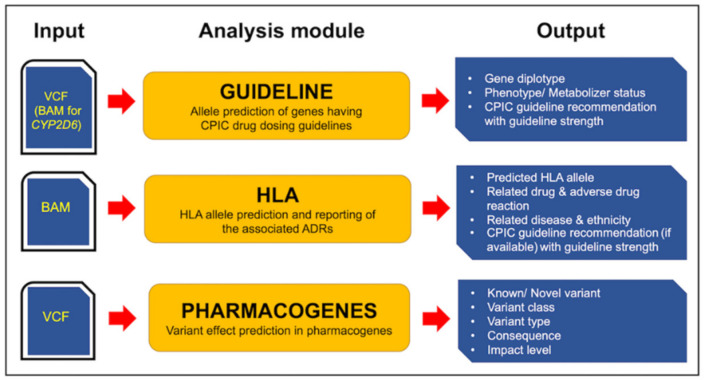
Overview of PharmVIP analysis modules. Software architecture of PharmVIP showing types of input files (VCF and BAM) and output report details from the three analytic modules, namely Guideline, HLA and Pharmacogenes.

We constructed a new PGx analysis tool (Figure 1), called PharmVIP, which offers three variant interpretation modules covering analyses of pharmacogenes involved in PK, PD, and IM-ADR. The first module is “Guideline”, which provides available CPIC drug dosing recommendations for relevant gene-drug pairs. Prediction of HLA genotypes and associated IM-ADRs are presented in the “HLA” module. Finally, the “Pharmacogenes” module offers effect prediction (gene function) from input variants in pharmacogenes. These three PGx modules cover PGx prediction for both “on target” and “off target” ADRs [25].

### 2.1. Starting a Project

**Figure 2:**
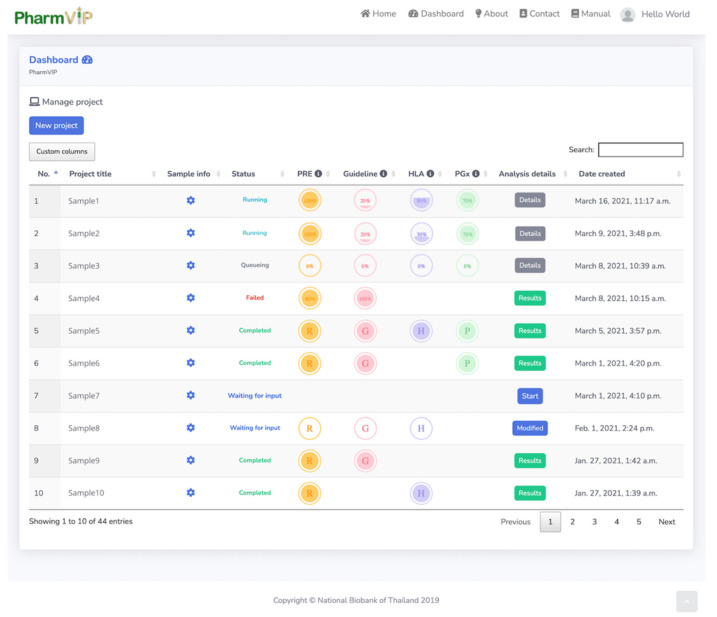
The Dashboard page summarizing the status and details of the projects. The current status of each project is shown in the column headed “Status”. After starting the analysis, the status changes from “queueing” to “running”, and the percentage of analysis progress is displayed for each module. PRE = Preprocessing step, Guideline = Guideline module, HLA = HLA module, PGx = Pharmacogenes module. When the analysis is finished, the output can be accessed through the “Results” button under the “Analysis details” column.

**Figure 3:**
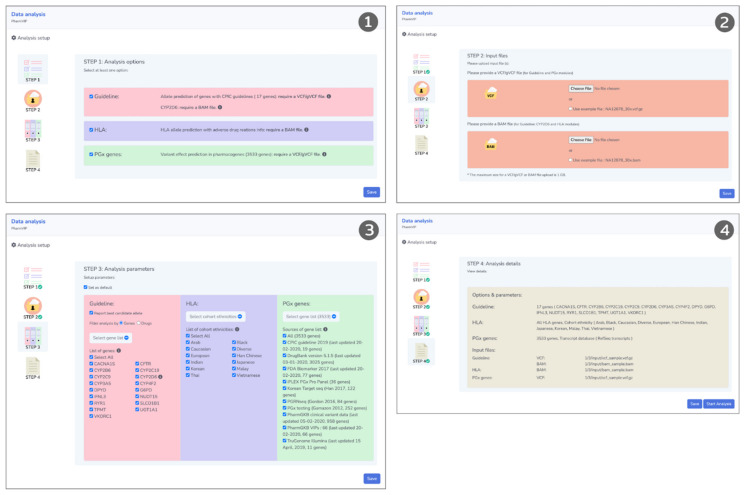
Four-step walkthrough of variant analysis. In step one, the user selects the desired analysis module(s). In step two, the user provides the corresponding input file(s). In step three, the user configures options for each of the selected analysis modules. In step four, the user verifies the chosen options before starting the analysis.

Due to the high demand for computational resources, PharmVIP requires registration for an account to prioritize and schedule the submission of PGx tasks. After the registration is confirmed, the account can be used to manage and create projects. Upon logging in, users can view the status and analysis reports of the available projects associated with the account. To start a new project, the user clicks on the ‘New project’ button on the dashboard page. The dashboard page will show the status of each project, i.e., waiting for an input, queueing, running, completed, or failed (Figure 2). For “running” analysis status, the percentage of analysis progress is also displayed. To perform data analysis, there are four steps that a user will be guided to follow. Step 1 prompts the user to choose at least one analytic module. Step 2 asks the user to upload the input files (either in VCF or BAM-file format) according to the requirements of the selected module(s). Step 3 asks the user to configure the selected modules via parameters that determine which pharmacogenes are analyzed and the prior cohort information for HLA analysis. Lastly, the user verifies the chosen configuration (Figure 3).

### 2.2. Guideline Module

The Guideline module is for allelic determination in pharmacogenes which have CPIC drug dosing guidelines. The guidelines are provided for the gene-drug pairs having strong associations between genotypes and drug response phenotypes. Currently, this Guideline module predicts alleles in 17 genes associated with 47 drugs. The module accepts input genotypic data in the VCF file format. If *CYP2D6* analysis is required, a BAM file of aligned NGS reads must also be inputted. From the input VCF file, the module identifies the diplotypes matched to the provided genotypic data. Then, the diplotypes are mapped to the relevant drug dosing guidelines, which are then reported. The input file size is limited to less than 1 GB due to the high demand for computational resources. For a larger input file, an in-house tool is provided (https://github.com/NBT-GeTH/pharmvip-filter (accessed on 15 November 2021)) for filtering the VCF file to retain only genotypic data of all pharmacogenes analyzed in PharmVIP and for filtering a BAM file for analysis of the *CYP2D6* gene region (as well as the *HLA* gene region for the HLA module). The Guideline module offers two analysis modes: analysis by gene list or by associated drug list. For the latter, drugs are grouped by the therapeutic category. Input genotypic data frequently contain unphased and missing variants which can create ambiguity for allele prediction. To decrease the volume of diplotype candidates arising from ambiguous allele prediction, users can set the module to report only the best candidate allele of each gene in the case that more than one allele per gene is predicted from the module.

### 2.3. HLA Module

The HLA module is for predicting HLA alleles and the reporting of the known adverse drug reactions (ADRs) associated with the identified HLA alleles and the relevant CPIC guideline recommendations. Currently, there are several NGS-based HLA genotyping software tools that are based on various algorithms and may yield different allele prediction outputs. By default, the module predicts alleles for all HLA genes from an input BAM file using three different tools (ATHLATES, HLA-HD, Kourami) and then reports the summarized results of all possible HLA alleles. We selected three different tools based on three different HLA genotyping approaches to be incorporated into the “HLA” module workflow. ATHLATES is a consensus-sequence-based typing method that assembles reads into contigs and subsequently matches the contigs against reference alleles [31]. HLA-HD relies on the read mapping to the reference allele repository and a score calculation based on weighted read counts for best allele selection [32]. Kourami utilizes a graph-guided assembly technique for constructing allele sequences given high-coverage WGS data [33]. The combined prediction results from all three methods inform users of the reliability of the predicted alleles. The allele prediction results are then checked against the HLA and Adverse Drug Reaction Database (HLA-ADR) [34] to identify associated ADRs. In case users wish to focus on ADRs that are known in specific populations, the module allows users to choose in advance specific ethnicities of the cohort with reported ADR cases associated with the HLA alleles.

### 2.4. Pharmacogenes Module

The Pharmacogenes module focuses on the identification of variants including SNPs, insertions, and deletions in all pharmacogenes from an input VCF file. The module then predicts the effects of these variants on gene function. The user can choose to analyze specific lists of pharmacogenes collated from various sources, i.e., publications, genotyping platforms, and databases such as PharmGKB.

### 2.5. Output Report

**Figure 4:**
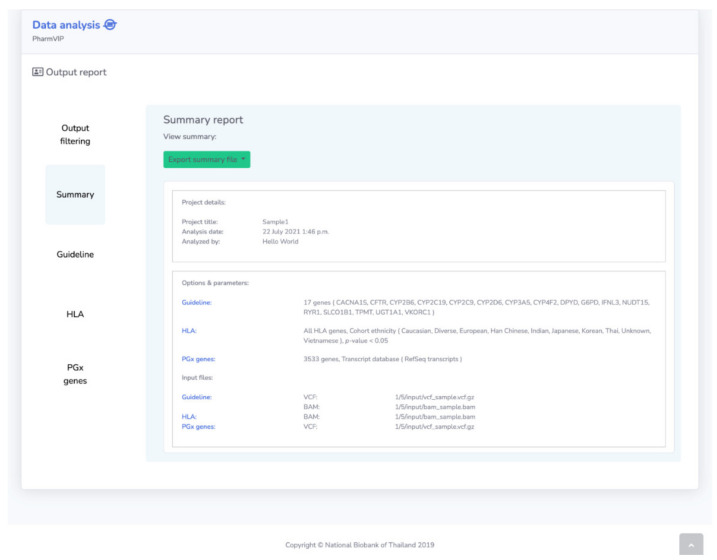
The output report page. The output report page consists of output filtering option(s), a summary report, and full reports of Guideline, HLA, and Pharmacogenes (PGx genes) modules.

After analyses from all selected modules are finished, the analysis outputs are generated and presented in a summary report of all modules, together with full reports of each module (Figure 4). The reports are presented in both an interactive web version and as exportable files.

#### 2.5.1. Summary Report

For the online interactive version, a summary report starts with the summary of the “Guideline” module which comprises two tables, the diplotype-matching table and the CPIC guideline assignment. The diplotype-matching table displays gene name, the phasing status of genotypic data, the number of missing variants, and the number and the list of matched diplotypes. Users can click on the link to see the details of each predicted diplotype that comprises the detail of each variant, including the rs-ID, the related allele of each variant, and the sample genotypic data. The read depth information, which indicates the number of reads supporting the assigned genotype, is also provided in this section for assessing the confidence of each called variant. In the second table, the summary file presents the CPIC guideline of the predicted diplotype, which includes the associated phenotype (metabolizer status) and the guideline strength. The gene and drug information from the PharmGKB database is also available as link-outs from the summary report. An example of a summary report in PDF format is provided in File S1 (Supplementary Materials).

Next, three output tables are displayed for the “HLA” module summary report (online interactive version). The first table is allele prediction, which reports predicted alleles of all HLA genes, the number of HLA prediction software tools that can detect such alleles, and the link to the HLA-ADR association studies that are related to the predicted HLA alleles. The second table shows the relevant CPIC guideline recommendations (if available) in the same format as shown in the “Guideline” module. The CPIC guideline related to an HLA gene and a gene in the “Guideline” module will also be displayed if both “Guideline” and “HLA” modules are selected for analysis. The third table gives the details of HLA-ADR association studies that are related to the predicted HLA alleles. It will show the details of the drug name, ADR, the disease of reported patients, the cohort ethnicity, the number of publications that reported these events, and the link to the full report of this module.

For the “Pharmacogenes” module, the summary is shown in the form of summary statistics of all identified variants, i.e., the frequency distribution of all variants identified in the sample described in several aspects including variant classes, variant types (novel/known), consequences (the effect of variants on gene function), impact level (high/moderate/low/modifier), and for missense variants, the effects on protein function (SIFT and Polyphen results).

#### 2.5.2. Full Report

**Figure 5:**
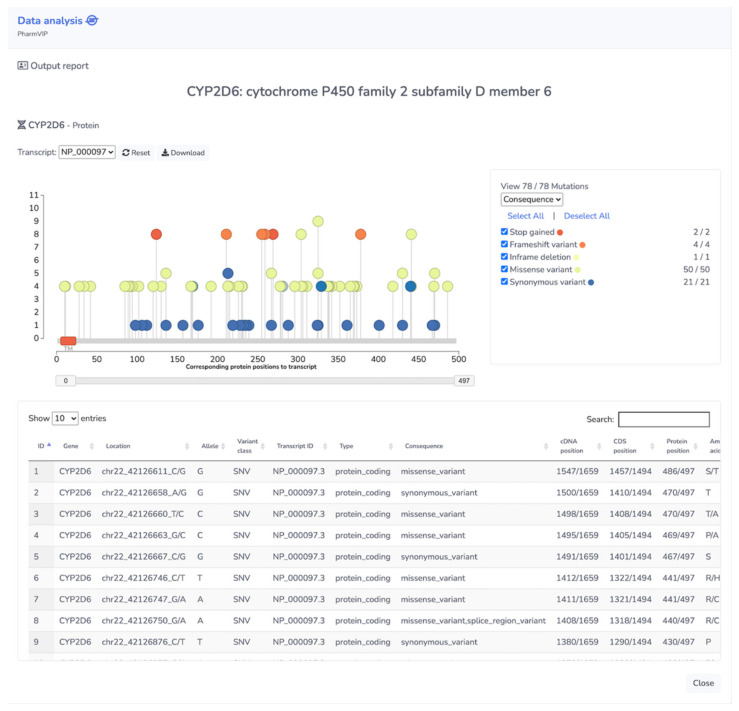
Lolliplot of the *CYP2D6* gene showing genetic variants. In the “gene” view option of the Pharmacogenes module, a lolliplot displays the locations of the 78 genetic variants along the *CYP2D6* gene region. The displayed variants can be filtered by variant consequences, which are illustrated by color code, and the variant impact levels, which are represented by the height of the plot on the *y*-axis (Low = 1, Moderate = 4, High = 8).

The full report of the “Guideline module” will show additional details of each CPIC drug dosing guideline. For the “HLA” module, it shows the same information as shown in the summary file plus the details of the HLA-ADR association study related to each predicted allele, including odds ratios and *p*-values of the reported HLA-ADR associations. There are also link-outs to the publications that report ADR cases. For the “Pharmacogenes” module full report, there are two view options, namely the “variant” view and the “gene” view. The “variant” view shows the list of all genetic variants with the details of variant consequences, which can be exported as an Excel file. For the “gene” view, the variants are grouped by genes and a lolliplot is displayed showing the locations of genetic variants along each gene region with their functional effects (Figure 5). This lolliplot of gene-level pharmacogenomic variants is useful for global visualization of variant effects on the function of each gene. Full report examples of all modules are provided in Files S2–File S4 (Supplementary Materials).

### 2.6. Post-Analysis Output Filtering

After checking the analysis outputs, there is an option to filter the results of each module separately to get a new analysis report comprising only the results of interest. For the “Guideline” module, the user can filter the results by gene/drug name, phenotype, and CPIC guideline strength. For example, after completing a default all-genes analysis, the user can obtain the CPIC guidelines relevant only to the *CYP2C19* gene and with strong supporting evidence for the guideline by using the output filtering option of the “Guideline” module. The “HLA” module allows result filtering by HLA genes and drugs/ADRs/patient diseases/cohort ethnicities of the HLA-ADR association studies. The “Pharmacogenes” module allows users to select only variants of interest by filtering variant classes, variant types, consequences, impact level, and SIFT/Polyphen classes.

### 2.7. Running Time

Typical run times on our PharmVIP web server for analyses of input VCF and BAM files (approximately ≤1 GB for each input file) in Guideline, HLA, and Pharmacogenes modules using the default parameters, are less than one minute, 12 min, and 25 min, respectively. All modules are run in parallel to minimize total run time.

### 2.8. PharmVIP Performance on Diplotype Assignment

To determine the accuracy of diplotype calling by the PharmVIP Guideline module, we compared the diplotype assignments of PharmVIP with those predicted from the PharmCAT tool [19] using publicly available data of 88 samples from the 1000 Genomes Project [35]. For an independent assessment of diplotype assignments made from NGS data, the consensus diplotype assignments from various pharmacogenetic testing assays employing non-NGS technologies on the same samples were used for comparison. These assignments were reported by the Centers for Disease Control and Prevention (CDC)—based Genetic Testing Reference Material Coordination (GeT-RM) Program [36]. The comparisons were performed for 11 pharmacogenes (*CYP2C19*, *CYP2C9*, *CYP2D6*, *CYP3A5*, *CYP4F2*, *DPYD*, *IFNL3*, *SLCO1B1*, *TPMT*, *UGT1A1*, and *VKORC1*) with diplotype assignments reported from all approaches (PharmVIP, PharmCAT, and GeT-RM). The diplotype assignments of PharmVIP were in perfect concordance with PharmCAT (Table 1, File S5 (Supplementary Materials)). This can be explained by the fact that allele matching methods implemented in PharmVIP and PharmCAT are based on similar algorithms and the same version of allele translation tables.

The concordance of diplotype assignment by PharmVIP and PharmCAT with GeT-RM was high for seven genes (*CYP2C19*, *CYP2C9*, *CYP3A5*, *CYP4F2*, *IFNL3*, *TPMT*, *VKORC1*), with an average of 98% concordance (Table 1). The diplotype assignments were in perfect concordance for *CYP2C9*, *IFNL3*, and *VKORC1*. For the *CYP2C19*, *CYP3A5*, *CYP4F2*, and *TPMT* genes, half of the discordant diplotypes (five of 10 cases) were displayed as “unknown allele” by PharmVIP (i.e., ?/?) and PharmCAT (i.e., not called), for which a consensus diplotype from GeT-RM is available (File S5). Since PharmVIP and PharmCAT prediction is based on CPIC allele definition tables of known alleles, any new diplotype that is not a combination of the known alleles will be labeled as unknown. To understand why the PharmVIP- and PharmCAT-reported diplotypes show low concordancy with the GeT-RM consensus for some genes, we inspected all discrepancies manually (File S5). Most of the discrepancies can be explained by different sets of variant positions used by GeT-RM (variants interrogated by the genotyping assays), different allele definitions used by GeT-RM assay platforms, and diplotype ambiguity for PharmVIP and PharmCAT owing to unphased NGS data. Further details of discordant diplotypes are described in File S6 (Supplementary Materials).

### 2.9. Feature Summary

The main purpose of developing PharmVIP was to provide a new data analysis tool for PGx researchers. PharmVIP provides three analysis/interpretation modules of pharmacogenomic variants through an easy-to-use web interface. Table 2 shows a PGx-tool feature comparison of Virtual Pharmacist, ePGA, PharmCAT, PharmaKU and PharmVIP. We did not compare PharmVIP with g-Nomic since it is a commercial tool. The main unique feature of PharmVIP is the HLA module which performs HLA allele prediction and reports the associated ADRs and relevant CPIC guidelines. Furthermore, the Pharmacogenes module in PharmVIP provides variant effect prediction results that are not available in other tools. The post-analysis output filtering option in PharmVIP is unique and allows users to tailor the report to fit their requirements without the need to rerun the analysis. By incorporating three modules and the underlying integrated workflows together in a parallel processing format, all analyses can be completed from just one input operation through a simple web interface. Moreover, PharmVIP provides a summary and a full output report for all modules, not only in an interactive web version but also as exportable files which can be utilized for further applications such as clinical practice in the future (for the “Guideline” and “HLA” module report).

### 2.10. Future Development

In the “Guideline” module, the read depth of each variant is displayed in the interactive web report, which can be used to assess the reliability of the assigned genotype. Future updates of PharmVIP will include a more explicit confidence value of the specified genotype based on other information such as the genotype quality. The “Guideline” module implementation in PharmVIP will be updated at regular intervals to provide drug dosing guidelines as much as up-to-date as possible. Population-specific phasing and imputation pipelines are planned to be included in order to increase the allele prediction accuracy in the future versions of PharmVIP.

For the “HLA” module, PharmVIP currently utilizes three HLA type inference algorithms from short-read sequencing data. The prediction accuracy of these algorithms relies on their core databases, which will be improved over time. We plan to collect more HLA types that represent southeast Asians to improve the HLA type inference from short reads. Furthermore, because HLA typing from long read sequencing platforms is becoming available, we plan to incorporate the HLA allele prediction for long-read sequencing data into the “HLA” module as well for improved accuracy of HLA typing.

It is noted that PharmVIP and other existing tools depend on the same publicly available knowledge bases in performing allele prediction and providing dosing guideline recommendations. The information in these knowledge bases does not represent all variants and the drug guidelines may be less accurate for certain populations. Allele translation tables and drug guidelines representing the distribution of variants observed in the Thai population are planned to be included in the future versions of PharmVIP.

## 3. Materials and Methods

**Figure 6:**
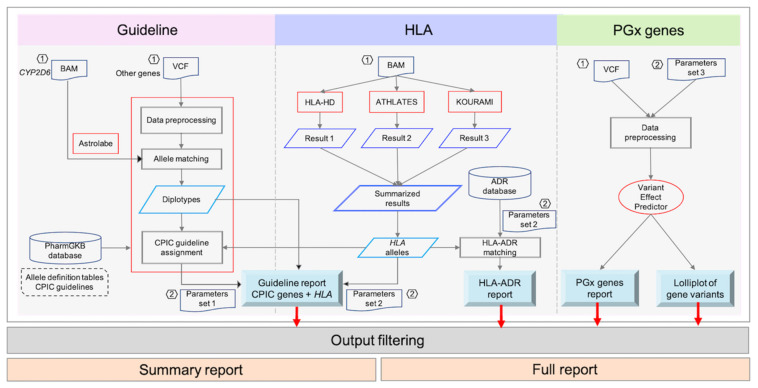
Overview workflows of the Guideline, HLA and Pharmacogenes analysis modules.

PharmVIP comprises three analysis modules: (1) Guideline, (2) HLA, and (3) Pharmacogenes. The workflows of the three modules are operated separately and the results from each are integrated (Figure 6). In general, PharmVIP accepts input variants in a standard VCF format (*.vcf), which can be produced by various workflows such as GATK [37,38], SpeedSeq [39], and DeepVariant [40]. To do HLA typing and *CYP2D6* allele prediction, PharmVIP requires a BAM file (*.bam), containing aligned NGS short reads to a human genome reference sequence (GRCh38). Since the quality of input files can affect the output results, the input file requirement and an example of data preprocessing workflow (to obtain the required BAM and VCF input files) are provided for users on the associated GitHub page (https://github.com/NBT-GeTH/pharmvip-guideline (accessed on 15 November 2021)).

### 3.1. Guideline Module

The workflow of the Guideline module comprises three main steps: (i) input data preprocessing, (ii) allele matching, and (iii) CPIC guideline assignment.

#### 3.1.1. Input Data Preprocessing

Allele definitions consisting of the allele names and their haplotype information of 11 pharmacogenes (*CFTR*, *CYP2C19*, *CYP2C9*, *CYP3A5*, *CYP4F2*, *DPYD*, *IFNL3*, *SLCO1B1*, *TPMT*, *UGT1A1*, and *VKORC1*) were downloaded from the PharmCAT GitHub page (release v0.6.0) [41]. These allele definitions are based on the PharmGKB allele definition tables [42] with some alterations (see PharmCAT website [43] for more information). Allele definition tables of the other five pharmacogenes (*CACNA1S*, *CYP2B6*, *G6PD*, *NUDT15*, *RYR1*) were downloaded from the PharmGKB database (downloaded on 20 February 2020) [13,42]. The Guideline module accepts an input VCF file, which is based on alignment to the GRCh38 reference genome. The sample genotypic data of the required haplotype positions (according to allele definition tables) of 16 pharmacogenes and other related information are parsed from the VCF data using cyvcf2 in bioconda package version 0.11.7 [44]. The VCF file gives information for determining the phasing status of each variant position. The phasing status for a variant position includes “phased”—phased variants or homozygous alleles, “unphased”—unphased variants which have heterozygous alleles, and “missing data”. The gene phasing status is determined from all variants in each pharmacogene where “phased-gene” is labeled when all of the gene’s variants are phased and “unphased-gene” when there exists at least one unphased-variant. An example of a VCF input file with the required phase information of each variant is provided in File S7 (Supplementary Materials).

#### 3.1.2. Allele Matching

For *CYP2D6* analysis, a BAM file is required for the allele (haplotype) prediction in addition to a VCF file. *CYP2D6* alleles are predicted using the default parameters of the Astrolabe tool [7]. Allele prediction for the other 16 pharmacogenes is performed using in-house scripts (https://github.com/NBT-GeTH/pharmvip-guideline (accessed on 15 November 2021)). The PharmGKB allele definition table is used to predict alleles from input genotypic data that will be converted into a python-regular-expression based on the variant phase status of a pharmacogene, which is determined from the previous data preprocessing step. For a phased gene, two regular expressions, one for each phased haplotype, are generated. Each pattern of the regular expression is compared with the entries from the allele definition table. A resolved diplotype is identified from two matched alleles (regular expressions). Multiple matched-diplotype candidates for a pharmacogene are reported when the gene contains one or more missing variants. The missing variant is treated as an ambiguous basecall matching any base. For an unphased gene, the genotype is converted to one or two regular expressions. If there are only unphased variants in an unphased gene, only one regular expression is generated, representing all possibilities of both haplotypes. For an unphased gene, all alleles in the allele definition table matched to the regular expression of the genotypes are identified. Then, all possible diplotypes are listed from any two alleles (haplotypes) that can be paired and matched with the sample genotypes. If there is no matched allele from the definition table, the result will be displayed as unknown (?/?), entailing a possible novel allele. For further investigation, the sample genotypic data and other related information of the unknown allele are provided in the diplotype-matching table on the summary report of the “Guideline” module. The presence of a recurring genotypic pattern found among several samples may suggest a possible novel allele. In the case of more than one diplotype candidate, users will be prompted to select the candidate allele for further analysis. The best allele selection approach is based on the PharmCAT method [19,45]. The special rules of allele assignment for *UGT1A1*, *SLCO1B1*, *CFTR*, and *DPYD* implemented in PharmCAT are also applied in PharmVIP. More details for the allele matching method with examples for all cases of sample genotypic data are provided in File S7.

#### 3.1.3. CPIC Dosing Guideline Assignment

This step takes the list of diplotype candidates obtained from the allele matching step and generates output reports comprising CPIC drug dosing recommendations. The data files required for CPIC drug dosing guideline assignment were downloaded using PharmGKB API version 1.0 (https://api.pharmgkb.org (accessed on 3 December 2019)). Using the API data, three files were generated that are used by PharmVIP in the guideline assignment process. The “allele name mapping” file maps an allele name from the allele definition table to the one used in the drug guideline. The “allele function mapping” file contains information of the function/metabolizer status of each allele for each gene-drug association. The “dosing guideline” file is used for the translation of allele function into the relevant guideline. For each matched diplotype from the allele matching step, the corresponding haplotype pair is mapped to the “allele name mapping” file for allele name conversion. Then, the function of each haplotype is determined from the “allele function mapping” file. Finally, the drug dosing guideline of each gene-drug pair is identified from the “dosing guideline” file based on the function combination of these two haplotypes.

### 3.2. HLA Module

A BAM file of sequence reads in FASTQ format aligned to the GRCh38 reference genome, is used as the input for three HLA genotyping tools: ATHLATES version 2014-04-26 [31], HLA-HD v1.2.0.1 [32], and Kourami v0.9.6 [33]. All tools utilize the HLA sequence library from the IPD-IMGT/HLA Database release 3.35.0 [46]. The preprocessing of an input BAM file by PharmVIP is tailored for the three tools as follows. Using Samtools v1.9 [47], a received BAM file is preprocessed to obtain only reads mapped to HLA-associated regions, which is then used as an input file for the Kourami tool. The BAM file is then processed further using the bam2FastQ function of bamUtil v1.0.14 [48] to convert the BAM file into FASTQ files, which are used as input for HLA-HD and ATHLATES tools. For each HLA genotyping tool, the analysis is performed using the tool’s default parameter settings. The three tools produce HLA allele prediction results that are collected and summarized using the in-house scripts (https://github.com/NBT-GeTH/pharmvip-hla (accessed on 15 November 2021)). PharmVIP suggests whether a predicted HLA type is associated with a known IM-ADR. This is done by constructing an internal data-warehouse which is a collection of the HLA and Adverse Drug Reaction Database (HLA-ADR) [34] (downloaded on 20 February 2020) and a prospective collection of known events found elsewhere. The relevant CPIC dosing recommendations associated with all predicted HLA alleles are also checked and reported in the “HLA” module using the same resources as utilized in the “Guideline” module.

### 3.3. Pharmacogenes Module

A total of 3533 pharmacogenes were collated from six main resources: (1) PharmGKB [13] comprising CPIC guidelines (downloaded on 20 February 2020), Very Important Pharmacogenes (downloaded on 20 February 2020), clinical variant data (downloaded on 5 February 2020); (2) US FDA comprising pharmacogenomic biomarkers in drug labeling [49] (downloaded on 20 February 2020); (3) pharmacogenomics-related publications: [6,8,50]; (4) PGx information from iPLEX PGx Pro Panel [51]; (5) information from TruGenome Pharmacogenomics Screen Test [52]; and (6) DrugBank [16].

Annotation of all pharmacogenes (based on GRCh38 version) was obtained from the UCSC Table Browser [53]. Tabix [54] was used to extract all pharmacogene-variants from an input VCF file. The predicted functional effects of single nucleotide polymorphisms (SNPs) and short INDELs (insertions and deletions) from these pharmacogenes are conducted using the Ensembl Variant Effect Predictor (VEP) platform version 95 [55] with the following parameters: --refseq --variant_class --sift b --polyphen b --protein --check_existing --total_length --pick --coding_only --no_intergenic. Only variants in protein-coding sequences are considered and only the most severe consequence for each variant is chosen with priority is given to the canonical transcript for each gene. A lolliplot is deployed to display information of variant consequences and impacts on the pharmacogene, using an in-house script modified from one available online [56]. The human protein domain information based on GRCh38 for generating lolliplots was downloaded from the HPRD Human Protein Reference Database [57,58].

### 3.4. System Design and Implementation

The PharmVIP web-application was constructed using Django, a high-level Python web framework, version 2.1.3 and Python3 version 3.7.4. All analysis modules were implemented using Python3 version 3.7.6., while the internal database services are provided by MySQL version 5.7.26. phpMyAdmin 4.9.3 was used to manage all the database builds. MinIO release 2020-01-16T03-05-44Z, a high-performance object storage platform, was deployed to provide read/write facilities for remote server storage activities. A deployment of PharmVIP was done as a container image using Docker 20.10.0 while Kubernetes 1.19.3 was used to manage computational resources.

### 3.5. Data Management

For security, privacy and economy purposes, the input data and the analysis results will be permanently removed from the server 10 days after the commencement of the analysis.

### 3.6. Evaluation of PharmVIP Performance on Diplotype Assignment

To investigate the performance of PharmVIP on diplotype assignment of pharmacogenes, we analyzed publicly available genotypic datasets of which there was already available genotyping results by pharmacogenetic testing assays from the Centers for Disease Control and Prevention (CDC)—based Genetic Testing Reference Material Coordination (GeT-RM) Program, as described previously [36]. In the GeT-RM study, 137 samples were genotyped by nine participating pharmacogenetic testing laboratories for 28 pharmacogenes using several commercially available and laboratory developed tests. The consensus diplotype results for all genes that were tested using two to six platforms were reported previously (in Supplemental Table S2 of Pratt et al. [36]). To start the PharmVIP analysis, the GRCh38 alignment files in CRAM format of whole genome sequencing data (30x coverage) of 88 (out of 137 GeT-RM study) samples, which are available in the 1000 Genomes Project, were downloaded from the 1000 Genomes Project data portal [35,59]. Variant calling was then performed on these alignment files using the Genome Analysis Toolkit (GATK) version 4.2 [60]. HaplotypeCaller was used to identify individual variants in GVCF format. Genotyping was done using the GenotypeGVCFs tool on the combined GVCF file. The individual GVCF file obtained was then used as an input for PharmVIP and PharmCAT (version 0.6.0) [19] which was operated with the default parameter settings. For each gene, the samples without the reported consensus diplotype information from GeT-RM were removed from consideration. The diplotype calling results obtained from PharmVIP were compared with the ones from PharmCAT analysis and the GeT-RM consensus diplotype data from PGx assays. The comparisons were performed on 11 pharmacogenes (*CYP2C9*, *CYP2C19*, *CYP2D6*, *CYP3A5*, *CYP4F2*, *DPYD*, *IFNL3*, *SLCO1B1*, *TPMT*, *UGT1A1*, *VKORC1*) with diplotype calls in PharmVIP, PharmCAT and GeT-RM. It should be noted that *IFNL3* was tested by only one PGx platform, there was no reported consensus and the *IFNL3* results from the non-consensus genotypes table (in Supplemental Table S3 of Pratt et al. [36]) were used for diplotype comparison.

## 4. Conclusions

PharmVIP is a one-stop PGx tool for the identification of variants/alleles in pharmacogenes and the interpretation of their consequences in terms of gene function, the associated ADRs, drug responses and drug dosing guidelines. Currently, the platform is in beta version and is provided for research and informational purposes to the PGx community and it is not meant to be a substitute for medical advice from a physician. Any comments/suggestions from users for improving the tool are welcome through the PharmVIP website (via the “Contact” menu) and through the associated GitHub page (https://github.com/NBT-GeTH (accessed on 10 November 2021)). This information will be utilized for tool evaluation and improvement in future versions of PharmVIP. PharmVIP is available via the URL: https://pharmvip.nbt.or.th (accessed on 15 November 2021).

## Figures and Tables

**Table 1:** Comparison of diplotype assignment results obtained from PharmVIP, PharmCAT, and GeT-RM.

Gene	Number of Concordant Samples/Total Number of Samples (% of Concordant Samples)		
	PharmVIP vs. GeT-RM	PharmCAT vs. GeT-RM	PharmVIP vs. PharmCAT
*CYP2C19*	85/88 (96.59%)	85/88 (96.59%)	88/88 (100.00%)
*CYP2C9*	88/88 (100.00%)	88/88 (100.00%)	88/88 (100.00%)
*CYP2D6*	35/88 (39.77%)	35/88 (39.77%)	88/88 (100.00%)
*CYP3A5*	87/88 (98.86%)	87/88 (98.86%)	88/88 (100.00%)
*CYP4F2*	59/64 (92.19%)	59/64 (92.19%)	64/64 (100.00%)
*DPYD*	47/88 (53.41%)	47/88 (53.41%)	88/88 (100.00%)
*IFNL3*	88/88 (100.00%)	88/88 (100.00%)	88/88 (100.00%)
*SLCO1B1*	20/88 (22.73%)	20/88 (22.73%)	88/88 (100.00%)
*TPMT*	87/88 (98.86%)	87/88 (98.86%)	88/88 (100.00%)
*UGT1A1*	42/64 (65.63%)	42/64 (65.63%)	64/64 (100.00%)
*VKORC1*	88/88 (100.00%)	88/88 (100.00%)	88/88 (100.00%)

**Table 2:** Feature comparison of available pharmacogenomic variant analysis tools and PharmVIP.

Features	Virtual Pharmacist	ePGA	PharmCAT	PharmaKU	PharmVIP
Input file	VCF, FASTQ, SNP array	VCF	VCF	VCF	VCF, BAM
Single variant-based analysis function ^1^	✓ ^2^	✓	×	×	✓
Haplotype analysis function	× ^2^	✓	✓	✓	✓
CPIC drug dosing guidelines	×	✓	✓	✓	✓
HLA allele prediction/HLA-ADR association/HLA-related CPIC guidelines	×	×	×	×	✓
Variant effect prediction	×	×	×	×	✓
Web-based user interface (for command line interface)	✓	✓	×	✓	✓
Exportable report files	✓	✓	✓	✓	✓
Gene-drug target network	✓	×	×	×	×
Post-analysis output filtering	×	×	×	×	✓
Updating of translation tables by users	×	✓	×	×	×
Group (multiple samples) analysis	✓	×	×	×	×
Installed as a local web server	✓	×	×	×	×
Choice of hg19 or hg38 as reference genome	×	×	×	✓	×

^1^ An analysis function that is intended to be performed on individual variants, such as the single variant translation based on PharmGKB clinical annotation data (genotype-based summaries describing the phenotypic impact of the variant) provided in Virtual Pharmacist and ePGA, and the effect prediction of single variant to the gene function provided in the PharmVIP Pharmacogenes module. ^2^ Tick mark denotes feature present/supported by the tool; cross mark denotes not present or not applicable.

## Data Availability

GitHub repository stores PharmVIP in-house scripts at https://github.com/NBT-GeTH (accessed on 10 November 2021).

## Supplementary Materials

The following are available online at https://www.mdpi.com/article/10.3390/jpm11111230/s1, File S1: An example of summary report, File S2: An example of full report of Guideline module, File S3: An example of full report of HLA module, File S4: An example of full report of Pharmacogenes module, File S5: Diplotype assignment results of GeT-RM, PharmVIP, and PharmCAT on 11 pharmacogenes, File S6: The details of discordant diplotypes between PharmVIP and the GeT-RM consensus, File S7: The details for the allele matching method of Guideline module.
