# Supplementary material for: PharmVIP: A Web-Based Tool for Pharmacogenomic Variant Analysis and Interpretation
Source: J Pers Med. 2021 Nov 19;11(11):1230. doi: 10.3390/jpm11111230 (PMC8618518; doi:10.3390/jpm11111230)
Supplement: Supplementary file 1 [file jpm-11-01230-s001.zip › File_S3.pdf]

HLA

Related CPIC guideline(s):

**Gene(s)**	**CYP2C9, HLA-B**
**All possible diplotype(s)**	**[*1/*2, Other/Other]**
**# missing variants (CYP2C9)**<br>**HLA-B**	**0/53**<br>**Tool(s) detecting Other/Other = ATHLATES,HLA-HD,KOURAMI**
**Drug: phenytoin**	
**Diplotype: [*1/*2, Other/Other]**	
Implications	Reduced phenytoin metabolism; higher plasma concentrations will increase probability of toxicities.
Metabolizers Status	N/A, N/A
Phenotype (Genotype)	An individual carrying one normal activity allele plus one decreased or no function allele.
Recommendations	Consider 25% reduction of recommended starting maintenance dosed (based on patient’s clinical characteristics). Subsequent maintenance doses should be adjusted according to therapeutic drug monitoring and response.
Guideline strength	Moderate
**Gene(s)**	**HLA-A, HLA-B**
**All possible diplotype(s)**	**[Other/Other, Other/Other]**
**HLA-A**<br>**HLA-B**	**Tool(s) detecting Other/Other = ATHLATES,HLA-HD,KOURAMI**<br>**Tool(s) detecting Other/Other = ATHLATES,HLA-HD,KOURAMI**
**Drug: carbamazepine**	
**Diplotype: [Other/Other, Other/Other]**	
Implications	Normal or reduced risk of carbamazepine-associated cutaneous adverse reactions
Metabolizers Status	N/A, N/A
Phenotype (Genotype)	"Normal" or reduced risk of carbamazepine-associated cutaneous adverse reactions in the absence of HLA-B*15:02 and HLA-A*31:01 alleles (often reported as "negative" on a genotyping test)
Recommendations	Use carbamazepine per standard dosing guidelines
Guideline strength	Strong

**Gene(s)**	**HLA-B**
**All possible diplotype(s)**	**Other/Other**
**HLA-B**	**Tool(s) detecting Other/Other = ATHLATES,HLA-HD,KOURAMI**
**Drug: abacavir**	
**Diplotype: Other/Other**	
Implications	Low or reduced risk of abacavir hypersensitivity
Metabolizers Status	N/A
Phenotype (Genotype)	Very low risk of hypersensitivity (~94% of patients) in the absence of *57:01 alleles (reported as "negative" on a genotyping test)
Recommendations	Use abacavir per standard dosing guidelines
Guideline strength	Strong
**Drug: allopurinol**	
**Diplotype: Other/Other**	
Implications	Low or reduced risk of allopurinol-induced SCAR
Metabolizers Status	N/A
Phenotype (Genotype)	Low or reduced risk of allopurinol SCAR (absence of *58:01 alleles (reported as "negative" on a genotyping test))
Recommendations	Use allopurinol per standard dosing guidelines.
Guideline strength	Strong
**Drug: oxcarbazepine**	
**Diplotype: Other/Other**	
Implications	Normal or reduced risk of oxcarbazepine-associated cutaneous adverse reactions
Metabolizers Status	N/A
Phenotype (Genotype)	"Normal" or reduced risk of oxcarbazepine-associated cutaneous adverse reactions in the absence of HLA-B*15:02 alleles (often reported as "negative" on a genotyping test)
Recommendations	Use oxcarbazepine per standard dosing guidelines
Guideline strength	Strong

Related publication(s) for HLA-ADR association:

HLA-A*01 (No. of detecting tools = 3/3)

ADR	Cohort ethnicity	Patient disease	p-value Patients and drug exposed controls	Odds ratio (95% confidence intervals) Patients and drug exposed controls	p-value Patients and population controls	Odds ratio (95% confidence intervals) Patients and population	Patient - carrier/allele frequency	Drug Exposed Controls - carrier/allele frequency	Population Controls - carrier/allele frequency	PMID
**Clozapine : 1 publication(s)**										
agranulocytosis	Caucasian	Schizophrenia					7 / 31	17 / 77		11266078

HLA-A*01:01 (No. of detecting tools = 3/3)

ADR	Cohort ethnicity	Patient disease	p-value Patients and drug exposed controls	Odds ratio (95% confidence intervals) Patients and drug exposed controls	p-value Patients and population controls	Odds ratio (95% confidence intervals) Patients and population	Patient - carrier/allele frequency	Drug Exposed Controls - carrier/allele frequency	Population Controls - carrier/allele frequency	PMID
**Amoxicillin-clavulanate : 2 publication(s)**										
drug induced liver injury (DILI)	Caucasian	Bacterial infections			1	0.76	35 / 134		37 / 107	21570397
drug induced liver injury (DILI)	Caucasian	Bacterial infections			1	0.38	3 / 134		21 / 107	21570397
**Antibacterial drugs : 2 publication(s)**										
Stevens–Johnson syndrome (SJS),toxic epidermal necrolysis (TEN),drug-induced hypersensitivity syndrome (DIHS),acute generalized exanthematous pustulosis (AGEP)	Unknown	Bacterial infections					3 / 24		7 / 80	31089385
Stevens–Johnson syndrome (SJS),toxic epidermal necrolysis (TEN),drug-induced hypersensitivity syndrome (DIHS),acute generalized exanthematous pustulosis (AGEP)	Unknown	Bacterial infections					2 / 20		7 / 80	31089385
**Antiepileptic drugs : 2 publication(s)**										
Stevens-Johnson syndrome (SJS),toxic epidermal necrolysis (TEN)	Han Chinese	Epilepsy	0.308	5.444 (0.311 - 95.21)			1 / 10	1 / 50		24856347
Stevens–Johnson syndrome (SJS),toxic epidermal necrolysis (TEN),drug-induced hypersensitivity syndrome (DIHS),acute generalized exanthematous pustulosis (AGEP)	Unknown	Epilepsy					5 / 34		7 / 80	31089385
**Carbamazepine : 5 publication(s)**										
Stevens-Johnson syndrome (SJS),toxic epidermal necrolysis (TEN),drug-induced hypersensitivity syndrome (DIHS)	Japanese	Epilepsy					0 / 61	6 / 376		21149285
Stevens-Johnson syndrome (SJS),toxic epidermal necrolysis (TEN)	Han Chinese	Epilepsy	0.33	6.14 (0.24 - 155.12)	0.21	5.65 (0.57 - 55.86)	1 / 36	0 / 64	3 / 528	22348435

**Carbamazepine : 5 publication(s)**										
Stevens-Johnson syndrome (SJS),toxic epidermal necrolysis (TEN)	Han Chinese	Epilepsy	1	3.13 (0.12 - 80.69)			1 / 24	0 / 24		22348435
Stevens-Johnson syndrome (SJS),toxic epidermal necrolysis (TEN)	European	Epilepsy			0.503	2.2 (1.1 - 4.7)	14 / 28		2731 / 8862	30972788
Stevens-Johnson syndrome (SJS),toxic epidermal necrolysis (TEN)	European	Epilepsy			0.06	3.3 (1.5 - 7)	14 / 28		124 / 529	30972788
**EGF receptor inhibitors : 1 publication(s)**										
maculopapular exanthema (MPE)	Unknown	Cancer	0.4008	1.446 (0.472 - 4.428)			4 / 38	25 / 172		23057547
**Lamotrigine : 2 publication(s)**										
Stevens–Johnson syndrome (SJS),toxic epidermal necrolysis (TEN)	Caucasian	Epilepsy	1	2 (0.04 - 78.77)	0.976	2.03 (0.07 - 29.33)	1 / 3	2 / 10	50 / 253	27888155
drug-induced hypersensitivity syndrome (DIHS)	Caucasian	Epilepsy	1	2 (0.04 - 78.77)	1	2.03 (0.07 - 29.33)	1 / 3	2 / 10	50 / 253	27888155
**Levetiracetam : 1 publication(s)**										
maculopapular eruption (MPE)	Indian	Epilepsy	1	0.58 (0.02 - 13.05)			0 / 10	3 / 50		28101480
**NSAID and 'multi-ingredient cold medication' : 1 publication(s)**										
Stevens-Johnson syndrome (SJS),toxic epidermal necrolysis (TEN),severe occular complicatoins	Diverse	Common cold			0.17		2 / 39		20 / 133	28278336

HLA-A*11 (No. of detecting tools = 3/3)

ADR	Cohort ethnicity	Patient disease	p-value Patients and drug exposed controls	Odds ratio (95% confidence intervals) Patients and drug exposed controls	p-value Patients and population controls	Odds ratio (95% confidence intervals) Patients and population	Patient - carrier/allele frequency	Drug Exposed Controls - carrier/allele frequency	Population Controls - carrier/allele frequency	PMID
**Tetanus antitoxin : 1 publication(s)**										
maculopapular eruption (MPE)	Han Chinese	Tetanus			0.12	0.24 (0.07 - 0.89)	3 / 15	/ 118	143 / 283	27749688

HLA-A*11:01 (No. of detecting tools = 2/3)

ADR	Cohort ethnicity	Patient disease	p-value Patients and drug exposed controls	Odds ratio (95% confidence intervals) Patients and drug exposed controls	p-value Patients and population controls	Odds ratio (95% confidence intervals) Patients and population	Patient - carrier/allele frequency	Drug Exposed Controls - carrier/allele frequency	Population Controls - carrier/allele frequency	PMID
**Amoxicillin-clavulanate : 2 publication(s)**										
drug induced liver injury (DILI)	Caucasian	Bacterial infections			0.26396	0.33	8 / 134		19 / 107	21570397
drug induced liver injury (DILI)	Caucasian	Bacterial infections			1	0.97	4 / 48		11 / 121	21570397

**Antibacterial drugs : 2 publication(s)**										
Stevens–Johnson syndrome (SJS),toxic epidermal necrolysis (TEN),drug-induced hypersensitivity syndrome (DIHS),acute generalized exanthematous pustulosis (AGEP)	Unknown	Bacterial infections			0.02		0 / 24		16 / 80	31089385
Stevens–Johnson syndrome (SJS),toxic epidermal necrolysis (TEN),drug-induced hypersensitivity syndrome (DIHS),acute generalized exanthematous pustulosis (AGEP)	Unknown	Bacterial infections					1 / 20		16 / 80	31089385
**Antiepileptic drugs : 1 publication(s)**										
Stevens–Johnson syndrome (SJS),toxic epidermal necrolysis (TEN),drug-induced hypersensitivity syndrome (DIHS),acute generalized exanthematous pustulosis (AGEP)	Unknown	Epilepsy			0.003		0 / 34		16 / 80	31089385
**Carbamazepine : 11 publication(s)**										
Stevens-Johnson syndrome (SJS),toxic epidermal necrolysis (TEN)	Han Chinese	Epilepsy		0.1 (0.05 - 0.2)			13 / 112	83 / 152		24268988
drug-induced hypersensitivity syndrome (DIHS)	Han Chinese	Epilepsy		1.9 (0.7 - 4.9)			16 / 23	83 / 152		24268988
maculopapular exanthema (MPE)	Han Chinese	Epilepsy		0.74 (0.4 - 1.4)			24 / 51	83 / 152		24268988
Stevens-Johnson syndrome (SJS),toxic epidermal necrolysis (TEN)	Han Chinese	Epilepsy		2.68 (1.4 - 5.1)			42 / 60	67 / 144		16538176
drug-induced hypersensitivity syndrome (DIHS)	Han Chinese	Epilepsy		0.99 (0.3 - 3)			6 / 13	67 / 144		16538176
maculopapular exanthema (MPE)	Han Chinese	Epilepsy		0.58 (0.2 - 1.6)			6 / 18	67 / 144		16538176
maculopapular exanthema (MPE)	Han Chinese	Epilepsy	0.931		0.769		22 / 40	28 / 52	37 / 72	23830818
Stevens-Johnson syndrome (SJS),toxic epidermal necrolysis (TEN),drug-induced hypersensitivity syndrome (DIHS)	Japanese	Epilepsy					7 / 61	68 / 376		21149285
Stevens-Johnson syndrome (SJS),toxic epidermal necrolysis (TEN)	Han Chinese	Epilepsy	0.54	0.75 (0.3 - 1.89)	0.86	1.07 (0.49 - 2.38)	9 / 36	22 / 64	141 / 528	22348435
Stevens-Johnson syndrome (SJS),toxic epidermal necrolysis (TEN)	Han Chinese	Epilepsy	0.38	0.6 (0.19 - 1.9)			9 / 24	12 / 24		22348435
Stevens–Johnson syndrome (SJS),toxic epidermal necrolysis (TEN)	Caucasian	Epilepsy	0.002	63.89 (2.46 - 45610000)	0.007	28.29 (1.42 - 180.77)	2 / 2	4 / 23	38 / 253	27888155
**Clarithromycin : 1 publication(s)**										
drug-induced skin reaction (DISI)	Han Chinese	Bacterial infections	0.38	4.55 (1.13 - 18.35)	7.22	1.47 (0.46 - 4.75)	7 / 12	8 / 34	138 / 283	29575644
**Lamotrigine : 2 publication(s)**										
maculopapular exanthema (MPE)	Han Chinese	Epilepsy	0.52		0.538		19 / 43	16 / 44	37 / 72	23830818
Stevens–Johnson syndrome (SJS),toxic epidermal necrolysis (TEN)	Caucasian	Epilepsy	0.395	10.5 (0.12 - 11530000)	0.784	2.83 (0.1 - 41.29)	1 / 3	0 / 10	38 / 253	27888155
**Levetiracetam : 1 publication(s)**										
maculopapular eruption (MPE)	Indian	Epilepsy	0.33	0.23 (0.02 - 2.36)			1 / 10	13 / 50		28101480

CONTACT
National Biobank of Thailand (NBT)
Tel : 025647000 Ext. 71475
Email : nbt@nstda.or.th

**NSAID and 'multi-ingredient cold medication' : 1 publication(s)**										
Stevens-Johnson syndrome (SJS),toxic epidermal necrolysis (TEN),severe occular complicatoins	Diverse	Common cold			0.00791	0.0743 (0.00438 - 1.26)	0 / 39		19 / 133	28278336
**Phenytoin : 2 publication(s)**										
Stevens-Johnson syndrome (SJS),toxic epidermal necrolysis (TEN)	Han Chinese	Epilepsy	0.3856	1.5 (0.6 - 3.6)			14 / 26	49 / 113		20235791
Stevens–Johnson syndrome (SJS),toxic epidermal necrolysis (TEN)	Caucasian	Epilepsy	1	1.04 (0.04 - 14.68)	1	0.71 (0.03 - 5.85)	1 / 2	3 / 28	38 / 253	27888155
**Tetanus antitoxin : 1 publication(s)**										
maculopapular eruption (MPE)	Han Chinese	Tetanus			0.21	0.26 (0.07 - 0.95)	3 / 15	/ 118	138 / 283	27749688

HLA-B*08:01 (No. of detecting tools = 3/3)

ADR	Cohort ethnicity	Patient disease	p-value Patients and drug exposed controls	Odds ratio (95% confidence intervals) Patients and drug exposed controls	p-value Patients and population controls	Odds ratio (95% confidence intervals) Patients and population	Patient - carrier/allele frequency	Drug Exposed Controls - carrier/allele frequency	Population Controls - carrier/allele frequency	PMID
**Amoxicillin-clavulanate : 2 publication(s)**										
drug induced liver injury (DILI)	Caucasian	Bacterial infections			1	0.68	28 / 134		31 / 107	21570397
drug induced liver injury (DILI)	Caucasian	Bacterial infections			1	0.49	3 / 48		16 / 121	21570397
**Antibacterial drugs : 2 publication(s)**										
Stevens–Johnson syndrome (SJS),toxic epidermal necrolysis (TEN),drug-induced hypersensitivity syndrome (DIHS),acute generalized exanthematous pustulosis (AGEP)	Unknown	Bacterial infections					0 / 24		2 / 80	31089385
Stevens–Johnson syndrome (SJS),toxic epidermal necrolysis (TEN),drug-induced hypersensitivity syndrome (DIHS),acute generalized exanthematous pustulosis (AGEP)	Unknown	Bacterial infections					1 / 20		2 / 80	31089385
**Antiepileptic drugs : 1 publication(s)**										
Stevens–Johnson syndrome (SJS),toxic epidermal necrolysis (TEN),drug-induced hypersensitivity syndrome (DIHS),acute generalized exanthematous pustulosis (AGEP)	Unknown	Epilepsy					4 / 34		2 / 80	31089385
**Carbamazepine : 3 publication(s)**										
Stevens-Johnson syndrome (SJS),toxic epidermal necrolysis (TEN),drug-induced hypersensitivity syndrome (DIHS)	Vietnamese	Epilepsy					0 / 38	1 / 25		25938071
Stevens-Johnson syndrome (SJS),toxic epidermal necrolysis (TEN)	European	Epilepsy			0.348	1.5 (0.7 - 3.5)	8 / 28		1835 / 8862	30972788
Stevens-Johnson syndrome (SJS),toxic epidermal necrolysis (TEN)	European	Epilepsy			0.113	2.1 (0.9 - 4.9)	8 / 28		85 / 529	30972788

**EGF receptor inhibitors : 1 publication(s)**										
maculopapular exanthema (MPE)	Unknown	Cancer	0.3929	0.638 (0.194 - 2.097)			4 / 38	12 / 172		23057547
**Lamotrigine : 1 publication(s)**										
drug-induced hypersensitivity syndrome (DIHS)	Caucasian	Epilepsy	0.114	12.6 (0.27 - 11530000)	0.716	3.22 (0.11 - 47.14)	1 / 3	0 / 10	34 / 253	27888155
**NSAID and 'multi-ingredient cold medication' : 1 publication(s)**										
Stevens-Johnson syndrome (SJS),toxic epidermal necrolysis (TEN),severe occular complicatoins	Diverse	Common cold			0.0483		1 / 39		20 / 133	28278336
**Phenytoin : 1 publication(s)**										
Stevens–Johnson syndrome (SJS),toxic epidermal necrolysis (TEN)	Caucasian	Epilepsy	1	1.63 (0.05 - 28.75)	1	0.81 (0.04 - 6.7)	1 / 2	2 / 28	34 / 253	27888155

HLA-B*56:01 (No. of detecting tools = 3/3)

ADR	Cohort ethnicity	Patient disease	p-value Patients and drug exposed controls	Odds ratio (95% confidence intervals) Patients and drug exposed controls	p-value Patients and population controls	Odds ratio (95% confidence intervals) Patients and population	Patient - carrier/allele frequency	Drug Exposed Controls - carrier/allele frequency	Population Controls - carrier/allele frequency	PMID
**Amoxicillin-clavulanate : 2 publication(s)**										
drug induced liver injury (DILI)	Caucasian	Bacterial infections			1	3.59	6 / 134		1 / 107	21570397
drug induced liver injury (DILI)	Caucasian	Bacterial infections			1	0.49	0 / 48		2 / 121	21570397
**Antibacterial drugs : 2 publication(s)**										
Stevens–Johnson syndrome (SJS),toxic epidermal necrolysis (TEN),drug-induced hypersensitivity syndrome (DIHS),acute generalized exanthematous pustulosis (AGEP)	Unknown	Bacterial infections					1 / 24		1 / 80	31089385
Stevens–Johnson syndrome (SJS),toxic epidermal necrolysis (TEN),drug-induced hypersensitivity syndrome (DIHS),acute generalized exanthematous pustulosis (AGEP)	Unknown	Bacterial infections					0 / 20		1 / 80	31089385
**Antiepileptic drugs : 1 publication(s)**										
Stevens–Johnson syndrome (SJS),toxic epidermal necrolysis (TEN),drug-induced hypersensitivity syndrome (DIHS),acute generalized exanthematous pustulosis (AGEP)	Unknown	Epilepsy					0 / 34		1 / 80	31089385
**Carbamazepine : 3 publication(s)**										
Stevens-Johnson syndrome (SJS),toxic epidermal necrolysis (TEN),drug-induced hypersensitivity syndrome (DIHS),maculopapular exanthema (MPE)	Japanese	Epilepsy	0.532	2.286 (0.133 - 39.203)			1 / 15	1 / 33		22211527
Stevens-Johnson syndrome (SJS),toxic epidermal necrolysis (TEN)	Han Chinese	Epilepsy	0.48	0.28 (0.01 - 7.67)			0 / 13	1 / 12		22348435
Stevens-Johnson syndrome (SJS),toxic epidermal necrolysis (TEN)	Thai	Epilepsy	0.3055	3.15 (0.31 - 31.62)			3 / 42	1 / 42		20345939

**NSAID and 'multi-ingredient cold medication' : 1 publication(s)**										
Stevens-Johnson syndrome (SJS),toxic epidermal necrolysis (TEN),severe occular complicatoins	Diverse	Common cold			0.575		0 / 39		4 / 133	28278336
**Oxcarbazepine : 1 publication(s)**										
maculopapular exanthema (MPE)	Han Chinese	Epilepsy	0.33	6.16 (0.24 - 156.27)	0.07	22.85 (2.01 - 259.72)	1 / 14	0 / 28	2 / 1236	23829937
**Phenytoin : 1 publication(s)**										
Stevens-Johnson syndrome (SJS),toxic epidermal necrolysis (TEN)	Han Chinese	Epilepsy	0.4654	2.2 (0.2 - 25.5)			1 / 26	2 / 113		20235791

HLA-C*01:02 (No. of detecting tools = 3/3)

ADR	Cohort ethnicity	Patient disease	p-value Patients and drug exposed controls	Odds ratio (95% confidence intervals) Patients and drug exposed controls	p-value Patients and population controls	Odds ratio (95% confidence intervals) Patients and population	Patient - carrier/allele frequency	Drug Exposed Controls - carrier/allele frequency	Population Controls - carrier/allele frequency	PMID
**Carbamazepine : 6 publication(s)**										
Stevens-Johnson syndrome (SJS),toxic epidermal necrolysis (TEN)	Han Chinese	Epilepsy	0.0086	0.26 (0.1 - 0.6)			8 / 60	54 / 144		16538176
drug-induced hypersensitivity syndrome (DIHS)	Han Chinese	Epilepsy		1.04 (0.3 - 3.2)			5 / 13	54 / 144		16538176
maculopapular exanthema (MPE)	Han Chinese	Epilepsy		1.67 (0.6 - 4.3)			9 / 18	54 / 144		16538176
Stevens-Johnson syndrome (SJS)	Korean	Epilepsy		2.1 (0.4 - 10.8)			3 / 7	13 / 50		21917426
drug-induced hypersensitivity syndrome (DIHS)	Korean	Epilepsy		0.6 (0.2 - 2.5)			3 / 17	13 / 50		21917426
drug-induced skin reaction (DISI)	Korean	Epilepsy		1.3 (0.4 - 4.2)			6 / 24	13 / 50		21917426
**Clarithromycin : 1 publication(s)**										
drug-induced skin reaction (DISI)	Han Chinese	Bacterial infections	0.21	5.4 (1.31 - 22.28)	0.73	3.05 (0.94 - 9.88)	7 / 12	7 / 34	89 / 283	29575644
**Lamotrigine : 1 publication(s)**										
maculopapular eruption (MPE)	Korean	Epilepsy	0.086	2.96 (0.92 - 9.53)	0.033	2.68 (1.11 - 6.5)	12 / 21	9 / 29	161 / 485	26282450
**NSAID and 'multi-ingredient cold medication' : 1 publication(s)**										
Stevens-Johnson syndrome (SJS),toxic epidermal necrolysis (TEN),severe occular complicatoins	Diverse	Common cold			0.686		1 / 39		8 / 133	28278336
**Phenytoin : 1 publication(s)**										
Stevens-Johnson syndrome (SJS),toxic epidermal necrolysis (TEN)	Han Chinese	Epilepsy	0.1103	0.4 (0.1 - 1.2)			5 / 26	41 / 113		20235791
**Tetanus antitoxin : 1 publication(s)**										
maculopapular eruption (MPE)	Han Chinese	Tetanus			0.09		8 / 15	/ 118	88 / 283	27749688

HLA-C*07 (No. of detecting tools = 3/3)

ADR	Cohort ethnicity	Patient disease	p-value Patients and drug exposed controls	Odds ratio (95% confidence intervals) Patients and drug exposed controls	p-value Patients and population controls	Odds ratio (95% confidence intervals) Patients and population	Patient - carrier/allele frequency	Drug Exposed Controls - carrier/allele frequency	Population Controls - carrier/allele frequency	PMID
**Clozapine : 1 publication(s)**										
agranulocytosis	Caucasian	Schizophrenia	0.02				20 / 31	30 / 77		11266078

HLA-C*07:01 (No. of detecting tools = 3/3)

ADR	Cohort ethnicity	Patient disease	p-value Patients and drug exposed controls	Odds ratio (95% confidence intervals) Patients and drug exposed controls	p-value Patients and population controls	Odds ratio (95% confidence intervals) Patients and population	Patient - carrier/allele frequency	Drug Exposed Controls - carrier/allele frequency	Population Controls - carrier/allele frequency	PMID
**EGF receptor inhibitors : 1 publication(s)**										
maculopapular exanthema (MPE)	Unknown	Cancer	0.1241	0.424 (0.176 - 1.023)			9 / 38	20 / 173		23057547
**Lamotrigine : 3 publication(s)**										
Stevens–Johnson syndrome (SJS),toxic epidermal necrolysis (TEN)	Caucasian	Epilepsy	0.846	4.5 (0.08 - 327.95)	1	1.26 (0.04 - 18.02)	1 / 3	1 / 10	72 / 253	27888155
drug-induced hypersensitivity syndrome (DIHS)	Caucasian	Epilepsy	0.217	18 (0.47 - 3603.25)	0.403	5.03 (0.35 - 142.38)	2 / 3	1 / 10	72 / 253	27888155
maculopapular eruption (MPE)	Korean	Epilepsy	0.254		1		0 / 21	3 / 29	3 / 485	26282450
**NSAID and 'multi-ingredient cold medication' : 1 publication(s)**										
Stevens-Johnson syndrome (SJS),toxic epidermal necrolysis (TEN),severe occular complicatoins	Diverse	Common cold			0.555		10 / 39		43 / 133	28278336
**Phenytoin : 2 publication(s)**										
Stevens–Johnson syndrome (SJS),toxic epidermal necrolysis (TEN)	Caucasian	Epilepsy	0.754	1.83 (0.26 - 12.6)	1	1.26 (0.24 - 5.86)	3 / 2	6 / 28	72 / 253	27888155
drug-induced hypersensitivity syndrome (DIHS)	Caucasian	Epilepsy	1	0.57 (0.02 - 7.71)	1	0.63 (0.03 - 6.11)	1 / 5	7 / 28	72 / 253	27888155

HLA-DPB1*04:01 (No. of detecting tools = 3/3)

ADR	Cohort ethnicity	Patient disease	p-value Patients and drug exposed controls	Odds ratio (95% confidence intervals) Patients and drug exposed controls	p-value Patients and population controls	Odds ratio (95% confidence intervals) Patients and population	Patient - carrier/allele frequency	Drug Exposed Controls - carrier/allele frequency	Population Controls - carrier/allele frequency	PMID
**Bucillamine : 1 publication(s)**										
bucillamine-induced proteinuria	Japanese	Rheumatoid arthritis		1.02			2 / 25	36 / 460		24899791

HLA-DPB1*14:01 (No. of detecting tools = 3/3)

ADR	Cohort ethnicity	Patient disease	p-value Patients and drug exposed controls	Odds ratio (95% confidence intervals) Patients and drug exposed controls	p-value Patients and population controls	Odds ratio (95% confidence intervals) Patients and population	Patient - carrier/allele frequency	Drug Exposed Controls - carrier/allele frequency	Population Controls - carrier/allele frequency	PMID
**Bucillamine : 1 publication(s)**										
bucillamine-induced proteinuria	Japanese	Rheumatoid arthritis		0.5			0 / 25	17 / 460		24899791

HLA-DQA1*01:01 (No. of detecting tools = 3/3)

ADR	Cohort ethnicity	Patient disease	p-value Patients and drug exposed controls	Odds ratio (95% confidence intervals) Patients and drug exposed controls	p-value Patients and population controls	Odds ratio (95% confidence intervals) Patients and population	Patient - carrier/allele frequency	Drug Exposed Controls - carrier/allele frequency	Population Controls - carrier/allele frequency	PMID
**Amoxicillin-clavulanate : 2 publication(s)**										
drug induced liver injury (DILI)	Caucasian	Bacterial infections			1	0.62	27 / 134		35 / 107	21570397
drug induced liver injury (DILI)	Caucasian	Bacterial infections			1	0.79	14 / 48		45 / 121	21570397

HLA-DQA1*05:01 (No. of detecting tools = 3/3)

ADR	Cohort ethnicity	Patient disease	p-value Patients and drug exposed controls	Odds ratio (95% confidence intervals) Patients and drug exposed controls	p-value Patients and population controls	Odds ratio (95% confidence intervals) Patients and population	Patient - carrier/allele frequency	Drug Exposed Controls - carrier/allele frequency	Population Controls - carrier/allele frequency	PMID
**Amoxicillin-clavulanate : 2 publication(s)**										
drug induced liver injury (DILI)	Caucasian	Bacterial infections			1	0.73	51 / 134		53 / 107	21570397
drug induced liver injury (DILI)	Caucasian	Bacterial infections			1	0.98	25 / 48		63 / 121	21570397

HLA-DQB1*02:01 (No. of detecting tools = 3/3)

ADR	Cohort ethnicity	Patient disease	p-value Patients and drug exposed controls	Odds ratio (95% confidence intervals) Patients and drug exposed controls	p-value Patients and population controls	Odds ratio (95% confidence intervals) Patients and population	Patient - carrier/allele frequency	Drug Exposed Controls - carrier/allele frequency	Population Controls - carrier/allele frequency	PMID
**Amoxicillin-clavulanate : 2 publication(s)**										
drug induced liver injury (DILI)	Caucasian	Bacterial infections			0.4724	0.55	42 / 134		49 / 107	21570397
drug induced liver injury (DILI)	Caucasian	Bacterial infections			1	0.64	19 / 48		61 / 121	21570397

**Clozapine : 1 publication(s)**										
agranulocytosis	Caucasian	Schizophrenia	0.07	2.22 (0.92 - 5.36)			13 / 30	20 / 77		11146763

HLA-DQB1*05:01 (No. of detecting tools = 3/3)

ADR	Cohort ethnicity	Patient disease	p-value Patients and drug exposed controls	Odds ratio (95% confidence intervals) Patients and drug exposed controls	p-value Patients and population controls	Odds ratio (95% confidence intervals) Patients and population	Patient - carrier/allele frequency	Drug Exposed Controls - carrier/allele frequency	Population Controls - carrier/allele frequency	PMID
**Amoxicillin-clavulanate : 2 publication(s)**										
drug induced liver injury (DILI)	Caucasian	Bacterial infections			1	0.65	23 / 134		30 / 107	21570397
drug induced liver injury (DILI)	Caucasian	Bacterial infections			1	0.69	10 / 48		36 / 121	21570397
**Bucillamine : 1 publication(s)**										
bucillamine-induced proteinuria	Japanese	Rheumatoid arthritis		0.77			3 / 25	69 / 460		24899791
**Oxcarbazepine : 1 publication(s)**										
maculopapular exanthema (MPE)	Korean	Epilepsy	0.254	0.23 (0.03 - 1.95)	0.012	0.13 (0.02 - 0.93)	1 / 40	7 / 70	82 / 485	27666425

HLA-DRB1*01 (No. of detecting tools = 3/3)

ADR	Cohort ethnicity	Patient disease	p-value Patients and drug exposed controls	Odds ratio (95% confidence intervals) Patients and drug exposed controls	p-value Patients and population controls	Odds ratio (95% confidence intervals) Patients and population	Patient - carrier/allele frequency	Drug Exposed Controls - carrier/allele frequency	Population Controls - carrier/allele frequency	PMID
**Flucloxacillin : 1 publication(s)**										
drug induced liver injury (DILI)	Caucasian	Bacterial infections	0.459				7 / 51	13 / 64		19483685

HLA-DRB1*01:01 (No. of detecting tools = 3/3)

ADR	Cohort ethnicity	Patient disease	p-value Patients and drug exposed controls	Odds ratio (95% confidence intervals) Patients and drug exposed controls	p-value Patients and population controls	Odds ratio (95% confidence intervals) Patients and population	Patient - carrier/allele frequency	Drug Exposed Controls - carrier/allele frequency	Population Controls - carrier/allele frequency	PMID
**Amoxicillin-clavulanate : 2 publication(s)**										
drug induced liver injury (DILI)	Caucasian	Bacterial infections			1	0.68	18 / 134		23 / 107	21570397
drug induced liver injury (DILI)	Caucasian	Bacterial infections			1	0.75	4 / 48		14 / 121	21570397
**Antiepileptic drugs : 1 publication(s)**										
Stevens-Johnson syndrome (SJS),toxic epidermal necrolysis (TEN)	Han Chinese	Epilepsy	0.167	10.2 (0.842 - 123.558)			1 / 10	0 / 50		24856347

**Bucillamine : 1 publication(s)**										
bucillamine-induced proteinuria	Japanese	Rheumatoid arthritis		0.83			3 / 25	65 / 460		24899791
**Carbamazepine : 2 publication(s)**										
maculopapular exanthema (MPE)	Han Chinese	Epilepsy	0.169		0.056		4 / 40	1 / 52	1 / 72	23830818
Stevens-Johnson syndrome (SJS),toxic epidermal necrolysis (TEN)	Han Chinese	Epilepsy	0.013	14 (1.53 - 128.1)			4 / 54	1 / 176		28476759
**Clozapine : 1 publication(s)**										
agranulocytosis	Caucasian	Schizophrenia	0.06				3 / 31	20 / 77		11266078
**Co-trimoxazole : 1 publication(s)**										
maculopapular exanthema (MPE),Stevens-Johnson syndrome (SJS)	Caucasian	HIV					11 / 53	12 / 83		19374512
**EGF receptor inhibitors : 1 publication(s)**										
maculopapular exanthema (MPE)	Unknown	Cancer	0.5782	0.812 (0.254 - 2.6)			4 / 38	15 / 173		23057547
**Nevirapine : 2 publication(s)**										
drug-induced hypersensitivity syndrome (DIHS)	Diverse	HIV	0.14				0 / 54	3 / 103		23328091
drug-induced skin reaction (DISI),drug induced liver injury (DILI)	Caucasian	HIV					0 / 26	3 / 72	7 / 164	16868443

HLA-DRB1*03 (No. of detecting tools = 3/3)

ADR	Cohort ethnicity	Patient disease	p-value Patients and drug exposed controls	Odds ratio (95% confidence intervals) Patients and drug exposed controls	p-value Patients and population controls	Odds ratio (95% confidence intervals) Patients and population	Patient - carrier/allele frequency	Drug Exposed Controls - carrier/allele frequency	Population Controls - carrier/allele frequency	PMID
**Flucloxacillin : 1 publication(s)**										
drug induced liver injury (DILI)	Caucasian	Bacterial infections	0.557				16 / 51	24 / 64		19483685

HLA-DRB1*03:01 (No. of detecting tools = 3/3)

ADR	Cohort ethnicity	Patient disease	p-value Patients and drug exposed controls	Odds ratio (95% confidence intervals) Patients and drug exposed controls	p-value Patients and population controls	Odds ratio (95% confidence intervals) Patients and population	Patient - carrier/allele frequency	Drug Exposed Controls - carrier/allele frequency	Population Controls - carrier/allele frequency	PMID
**Allopurinol : 1 publication(s)**										
Stevens-Johnson syndrome (SJS),toxic epidermal necrolysis (TEN),drug-induced hypersensitivity syndrome (DIHS)	Han Chinese	Gout	0.0000028	12.7	0.00085	10.3	33 / 51	17 / 135	14 / 93	15743917
**Amoxicillin-clavulanate : 2 publication(s)**										
drug induced liver injury (DILI)	Caucasian	Bacterial infections			1	0.71	30 / 134		31 / 107	21570397

**Amoxicillin-clavulanate : 2 publication(s)**										
drug induced liver injury (DILI)	Caucasian	Bacterial infections			1	0.83	10 / 48		29 / 121	21570397
**Carbamazepine : 1 publication(s)**										
maculopapular exanthema (MPE)	Han Chinese	Epilepsy	0.024		0.75		3 / 40	14 / 52	8 / 72	23830818
**Clozapine : 1 publication(s)**										
agranulocytosis	Caucasian	Schizophrenia					10 / 31	14 / 77		11266078
**Co-trimoxazole : 1 publication(s)**										
maculopapular exanthema (MPE),Stevens-Johnson syndrome (SJS)	Caucasian	HIV					13 / 53	17 / 83		19374512
**Nevirapine : 1 publication(s)**										
drug-induced hypersensitivity syndrome (DIHS)	Diverse	HIV	0.086				4 / 54	15 / 103		23328091
**Phenytoin : 1 publication(s)**										
Stevens-Johnson syndrome (SJS),toxic epidermal necrolysis (TEN)	Han Chinese	Epilepsy	0.4618	0.3 (0 - 2.7)			1 / 26	12 / 113		20235791

HLA-DRB3*01:01 (No. of detecting tools = 3/3)

ADR	Cohort ethnicity	Patient disease	p-value Patients and drug exposed controls	Odds ratio (95% confidence intervals) Patients and drug exposed controls	p-value Patients and population controls	Odds ratio (95% confidence intervals) Patients and population	Patient - carrier/allele frequency	Drug Exposed Controls - carrier/allele frequency	Population Controls - carrier/allele frequency	PMID
**Clozapine : 1 publication(s)**										
agranulocytosis	Caucasian	Schizophrenia					10 / 31	19 / 77		11266078

CONTACT
National Biobank of Thailand (NBT)
Tel : 025647000 Ext. 71475
Email : nbt@nstda.or.th

DISCLAIMER:

PharmVIP platform is currently provided for research and informational purposes only and is not intended to diagnose, treat, cure, or prevent any disease. The report generated by the platform is not meant to be a substitute for professional medical advice, diagnosis, or treatment provided by a physician or other qualified health care professional. Only a physician, pharmacist or other healthcare professional should advise a patient on the use of the medications prescribed. Any application of the content provided or obtained through the use of the platform is therefore solely at the user's own risk and responsibility.
