# Supplementary material for: PharmVIP: A Web-Based Tool for Pharmacogenomic Variant Analysis and Interpretation
Source: J Pers Med. 2021 Nov 19;11(11):1230. doi: 10.3390/jpm11111230 (PMC8618518; doi:10.3390/jpm11111230)
Supplement: Supplementary file 1 [file jpm-11-01230-s001.zip › File_S2.pdf]

Guideline

Related CPIC guideline(s):

**Gene(s)**	**CACNA1S, RYR1**
**All possible diplotype(s)**	**[Reference/Reference, Reference/Reference]**
**# missing variants (CACNA1S)**	**0/2**
**# missing variants (RYR1)**	**0/45**
**Drug: desflurane, enflurane, halothane, isoflurane, methoxyflurane, sevoflurane, succinylcholine**	
**Diplotype: [Reference/Reference, Reference/Reference]**	
Implications	These results do not eliminate the chance that this patient is susceptible to Malignant Hyperthermia (MH). The genetic cause of about half of all MH survivors, with MH susceptibility confirmed by contracture test, remains unknown [Article:28902675].
Metabolizer Status	N/A, N/A
Phenotype (Genotype)	An individual negative for a *RYR1* or *CACNA1S* malignant hyperthermia causative variant as designated by the European Malignant Hyperthermia Group (EMHG).*A negative or inconclusive genetic test cannot be assumed to indicate normal RYR1-related phenotype and should be interpreted in context of clinical findings, family history and other laboratory data. It is recognized that clinical laboratories and treating physicians can make a determination that a variant not evaluated by EMHG is pathogenic. EMHG website https://www.emhg.org/diagnostic-mutations accessed September 8, 2018.*
Recommendations	Clinical findings, family history, further genetic testing and other laboratory data should guide use of halogenated volatile anesthetics or depolarizing muscle relaxants.
Guideline strength	Strong

**Gene(s)**	**CFTR**
**All possible diplotype(s)**	**No CPIC variants found**
**# missing variants (CFTR)**	**0/54**
**Drug: ivacaftor**	
**Diplotype: No CPIC variants found**	
Implications	N/A
Metabolizer Status	N/A
Phenotype (Genotype)	N/A
Recommendations	This guideline does not contain recommendations for this allele combination.
Guideline strength	N/A

CONTACT
National Biobank of Thailand (NBT)
Tel : 025647000 Ext. 71475
Email : nbt@nstda.or.th

**Gene(s)**	**CYP2B6**
**All possible diplotype(s)**	***1/*1**
**# missing variants (CYP2B6)**	**1/35**
**Drug: efavirenz**	
**Diplotype: *1/*1**	
Implications	Normal efavirenz metabolism.
Metabolizer Status	Normal Metabolizer
Phenotype (Genotype)	An individual carrying two normal function alleles.
Recommendations	Initiate efavirenz with standard dosing (600 mg/day).
Guideline strength	Strong
**Gene(s)**	**CYP2C19**
**All possible diplotype(s)**	***1/*2**
**# missing variants (CYP2C19)**	**2/34**
**Drug: citalopram, escitalopram**	
**Diplotype: *1/*2**	
Implications	Reduced metabolism when compared to normal metabolizers.
Metabolizer Status	Intermediate Metabolizer
Phenotype (Genotype)	An individual carrying one normal function allele or one increased function allele and one no function allele.*The predicted metabolizer phenotype for the*2/*17 genotypes is a provisional classification. The currently available evidence indicates that the CYP2C19*17 increased function allele is unable to completely compensate for the no function CYP2C19*2.*
Recommendations	Initiate therapy with recommended starting dose.
Guideline strength	Strong
**Drug: clopidogrel**	
**Diplotype: *1/*2**	
Implications	Reduced platelet inhibition; increased residual platelet aggregation; increased risk for adverse cardiovascular events
Metabolizer Status	Intermediate Metabolizer
Phenotype (Genotype)	An individual carrying one normal function allele and one no function allele or one increased function allele and no function allele.*The predicted metabolizer phenotype for *2-*8/*17 genotypes are provisional classifications. The currently available evidence indicates that the *17 increased function allele is unable to completely compensate for the *2 no function allele [Article:20492469]; however, this data has not been consistently replicated and is therefore a provisional classification.*
Recommendations	Alternative antiplatelet therapy (if no contraindication); e.g., prasugrel, ticagrelor

CONTACT
National Biobank of Thailand (NBT)
Tel : 025647000 Ext. 71475
Email : nbt@nstda.or.th

**Drug: clopidogrel**	
**Diplotype: *1/*2**	
Guideline strength	Moderate
**Drug: sertraline**	
**Diplotype: *1/*2**	
Implications	Reduced metabolism when compared to normal metabolizers.
Metabolizer Status	Intermediate Metabolizer
Phenotype (Genotype)	An individual carrying one normal function allele or one increased function allele and one no function allele.*The predicted metabolizer phenotype for the*2/*17 genotypes is a provisional classification. The currently available evidence indicates that the CYP2C19*17 increased function allele is unable to completely compensate for the no function CYP2C19*2.*
Recommendations	Initiate therapy with recommended starting dose.
Guideline strength	Strong
**Drug: voriconazole**	
**Diplotype: *1/*2**	
Implications	Higher dose-adjusted trough concentrations of voriconazole compared to normal metabolizers.
Metabolizer Status	Intermediate Metabolizer
Phenotype (Genotype)	An individual carrying one normal function allele and one no function allele or one no function allele and one increased function allele.*The predicted metabolizer phenotype for the *2/*17 genotype is a provisional classification. The currently available evidence indicates that the CYP2C19*17 increased function allele is unable to completely compensate for the no function CYP2C19*2 (Sibbing et al., 2010). See the 2016 Supplement for a more comprehensive list of predicted metabolizer phenotypes.*
Recommendations	For pediatric or adult patients: initiate therapy with recommended standard of care dosing.*Further dose adjustments or selection of alternative therapy may be necessary due to other clinical factors, such as drug interactions, hepatic function, renal function, species, site of infection, TDM, and comorbidities.*
Guideline strength	Moderate

CONTACT
National Biobank of Thailand (NBT)
Tel : 025647000 Ext. 71475
Email : nbt@nstda.or.th

**Gene(s)**	**CYP2C19, CYP2D6**
**All possible diplotype(s)**	**[*1/*2, *3/*4]**
**# missing variants (CYP2C19)**	**2/34**
**CYP2D6**	**Analyzed by astrolabe**
**Drug: amitriptyline**	
**Diplotype: [*1/*2, *3/*4]**	
Implications	**For CYP2D6:**Greatly reduced metabolism of TCAs to less active compounds compared to normal metabolizers. Higher plasma concentrations of active drug will increase the probability of side effects.<br>**For CYP2C19:**Reduced metabolism of tertiary amines compared to normal metabolizers.
Metabolizer Status	Intermediate Metabolizer, Poor Metabolizer
Phenotype (Genotype)	**For CYP2D6:**An individual carrying only no function alleles.<br>**For CYP2C19:**An individual carrying one normal and one no function allele or one no and one increased function allele.
Recommendations	Avoid amitriptyline use. If amitriptyline is warranted, consider 50% reduction of recommended starting dose.*Utilizing therapeutic drug monitoring if a tricyclic is prescribed to a patient with CYP2D6 ultrarapid, intermediate or poor metabolism in combination with CYP2C19 ultrarapid, intermediate or poor metabolism is strongly recommended. Patients may receive an initial low dose of TCAs, which is then increased over several days to the recommended steady-state dose. The starting dose in this guideline refers to the recommended steady-state dose. Dosing recommendations only apply to higher initial doses of TCAs for treatment of conditions such as depression.*
Guideline strength	Optional
**Drug: clomipramine**	
**Diplotype: [*1/*2, *3/*4]**	
Implications	**For CYP2D6:**Greatly reduced metabolism of TCAs to less active compounds compared to normal metabolizers. Higher plasma concentrations of active drug will increase the probability of side effects.<br>**For CYP2C19:**Reduced metabolism of tertiary amines compared to normal metabolizers.
Metabolizer Status	Intermediate Metabolizer, Poor Metabolizer
Phenotype (Genotype)	**For CYP2D6:**An individual carrying only no function alleles.<br>**For CYP2C19:**An individual carrying one normal and one no function allele or one no and one increased function allele.
Recommendations	Avoid amitriptyline use. If amitriptyline is warranted, consider 50% reduction of recommended starting dose.*Utilizing therapeutic drug monitoring if a tricyclic is prescribed to a patient with CYP2D6 ultrarapid, intermediate or poor metabolism in combination with CYP2C19 ultrarapid, intermediate or poor metabolism is strongly recommended. Patients may receive an initial low dose of TCAs, which is then increased over several days to the recommended steady-state dose. The starting dose in this guideline refers to the recommended steady-state dose. Dosing recommendations only apply to higher initial doses of TCAs for treatment of conditions such as depression.*
Guideline strength	Optional

**Drug: doxepin**	
**Diplotype: [*1/*2, *3/*4]**	
Implications	**For CYP2D6:**Greatly reduced metabolism of TCAs to less active compounds compared to normal metabolizers. Higher plasma concentrations of active drug will increase the probability of side effects.<br>**For CYP2C19:**Reduced metabolism of tertiary amines compared to normal metabolizers.
Metabolizer Status	Intermediate Metabolizer, Poor Metabolizer
Phenotype (Genotype)	**For CYP2D6:**An individual carrying only no function alleles.<br>**For CYP2C19:**An individual carrying one normal and one no function allele or one no and one increased function allele.
Recommendations	Avoid amitriptyline use. If amitriptyline is warranted, consider 50% reduction of recommended starting dose.*Utilizing therapeutic drug monitoring if a tricyclic is prescribed to a patient with CYP2D6 ultrarapid, intermediate or poor metabolism in combination with CYP2C19 ultrarapid, intermediate or poor metabolism is strongly recommended. Patients may receive an initial low dose of TCAs, which is then increased over several days to the recommended steady-state dose. The starting dose in this guideline refers to the recommended steady-state dose. Dosing recommendations only apply to higher initial doses of TCAs for treatment of conditions such as depression.*
Guideline strength	Optional

**Drug: imipramine**	
**Diplotype: [*1/*2, *3/*4]**	
Implications	**For CYP2D6:**Greatly reduced metabolism of TCAs to less active compounds compared to normal metabolizers. Higher plasma concentrations of active drug will increase the probability of side effects.<br>**For CYP2C19:**Reduced metabolism of tertiary amines compared to normal metabolizers.
Metabolizer Status	Intermediate Metabolizer, Poor Metabolizer
Phenotype (Genotype)	**For CYP2D6:**An individual carrying only no function alleles.<br>**For CYP2C19:**An individual carrying one normal and one no function allele or one no and one increased function allele.
Recommendations	Avoid amitriptyline use. If amitriptyline is warranted, consider 50% reduction of recommended starting dose.*Utilizing therapeutic drug monitoring if a tricyclic is prescribed to a patient with CYP2D6 ultrarapid, intermediate or poor metabolism in combination with CYP2C19 ultrarapid, intermediate or poor metabolism is strongly recommended. Patients may receive an initial low dose of TCAs, which is then increased over several days to the recommended steady-state dose. The starting dose in this guideline refers to the recommended steady-state dose. Dosing recommendations only apply to higher initial doses of TCAs for treatment of conditions such as depression.*
Guideline strength	Optional

**Drug: trimipramine**	
**Diplotype: [*1/*2, *3/*4]**	
Implications	**For CYP2D6:**Greatly reduced metabolism of TCAs to less active compounds compared to normal metabolizers. Higher plasma concentrations of active drug will increase the probability of side effects.<br>**For CYP2C19:**Reduced metabolism of tertiary amines compared to normal metabolizers.
Metabolizer Status	Intermediate Metabolizer, Poor Metabolizer
Phenotype (Genotype)	**For CYP2D6:**An individual carrying only no function alleles.<br>**For CYP2C19:**An individual carrying one normal and one no function allele or one no and one increased function allele.
Recommendations	Avoid amitriptyline use. If amitriptyline is warranted, consider 50% reduction of recommended starting dose.*Utilizing therapeutic drug monitoring if a tricyclic is prescribed to a patient with CYP2D6 ultrarapid, intermediate or poor metabolism in combination with CYP2C19 ultrarapid, intermediate or poor metabolism is strongly recommended. Patients may receive an initial low dose of TCAs, which is then increased over several days to the recommended steady-state dose. The starting dose in this guideline refers to the recommended steady-state dose. Dosing recommendations only apply to higher initial doses of TCAs for treatment of conditions such as depression.*
Guideline strength	Optional

**Gene(s)**	**CYP2C9, CYP4F2, VKORC1**
**All possible diplotype(s)**	**[*1/*2, *1/*1, No info]**
**# missing variants (CYP2C9)**	**0/53**
**# missing variants (CYP4F2)**	**0/2**
**# missing variants (VKORC1)**	**1/1**

Drug: warfarin

Diplotype: [*1/*2, *1/*1, No info]

No Guideline.

CONTACT
National Biobank of Thailand (NBT)
Tel : 025647000 Ext. 71475
Email : nbt@nstda.or.th

**Gene(s)**	**CYP2D6**
**All possible diplotype(s)**	***3/*4**
**CYP2D6**	**Analyzed by astrolabe**
**Drug: atomoxetine**	
**Diplotype: *3/*4**	
Implications	Significantly decreased metabolism of atomoxetine may result in higher concentrations as compared to non-poor metabolizers. This may increase the occurrence of treatment-emergent side effects, but also a greater improvement of ADHD symptoms as compared to non-poor metabolizers in those who tolerate treatment. Poor metabolizer status is associated with lower final dose requirements as compared to non-poor metabolizers.
Metabolizer Status	Poor Metabolizer
Phenotype (Genotype)	An individual carrying only no functional alleles.
Recommendations	**Children:**<br>Initiate with a dose of 0.5 mg/kg/day and if no clinical response and in the absence of adverse events after 2 weeks, consider obtaining a plasma concentration 4 h after dosing. If response is inadequate and concentration is <200 ng/ml, consider a proportional dose increase to achieve a concentration to approach 400 ng/ml. If unacceptable side effects are present at any time, consider a reduction in dose.<br>**Strength of recommendation:** Strong<br>**Adults:**<br>Initiate with a dose of 40 mg/day and if no clinical response and in the absence of adverse events after 2 weeks increase dose to 80 mg/day. If response is inadequate after 2 weeks consider obtaining a plasma concentration 2-4 h after dosing. If concentration is <200 ng/ml, consider a proportional dose increase to achieve a concentration to approach 400 ng/ml. If unacceptable side effects are present at any time, consider a reduction in dose.<br>**Strength of recommendation:** Moderate<br>*Children and Adults:*<br>*Therapeutic range of 200 to 1000 ng/ml has been proposed [Article:29493375]. Limited data are available regarding the relationship between atomoxetine plasma concentrations and clinical response. Available information suggests that clinical response is greater in PMs compared to non-PMs and may be related to the higher plasma concentrations 1 to 1.5 hours after dosing in PMs compared to non-PMs administered a similar dose. Furthermore, modest improvement in response, defined as reduction in ADHD-RS, is observed at peak concentrations greater than 400 ng/ml.*
Guideline strength	Moderate
**Drug: codeine**	
**Diplotype: *3/*4**	
Implications	Greatly reduced morphine formation following codeine administration, leading to insufficient pain relief.
Metabolizer Status	Poor Metabolizer
Phenotype (Genotype)	An individual carrying no functional alleles.

CONTACT
National Biobank of Thailand (NBT)
Tel : 025647000 Ext. 71475
Email : nbt@nstda.or.th

**Drug: codeine**	
**Diplotype: *3/*4**	
Recommendations	Avoid codeine use due to lack of efficacy.*Considerations for alternative opioids: Alternatives that are not affected by this CYP2D6 phenotype include morphine and non-opioid analgesics. Tramadol, and to a lesser extent hydrocodone and oxycodone, are not good alternatives because their metabolism is affected by CYP2D6 activity; these agents should be avoided. There is substantial evidence for decreased efficacy of tramadol in poor metabolizers and a single case report of toxicity in an ultrarapid metabolizer with renal impairment following tramadol post-surgery. Use of other analgesics in CYP2D6 poor and ultrarapid metabolizers may therefore be preferable. Some other opioid analgesics are metabolized by CYP2D6, such as hydrocodone and oxycodone. To avoid treatment complications, opioids that are not metabolized by CYP2D6, including morphine, oxymorphone, buprenorphine, fentanyl, methadone and hydromorphone, along with non-opioid analgesics, may be considered as alternatives for use in CYP2D6 poor and ultrarapid metabolizers.*
Guideline strength	Strong
**Drug: desipramine**	
**Diplotype: *3/*4**	
Implications	Greatly reduced metabolism of TCAs to less active compounds compared to normal metabolizers. Higher plasma concentrations of active drug will increase the probability of side effects.
Metabolizer Status	Poor Metabolizer
Phenotype (Genotype)	An individual carrying only no function alleles.
Recommendations	Avoid tricyclic use due to potential for side effects. Consider alternative drug not metabolized by CYP2D6.If a TCA is warranted, consider 50% reduction of recommended starting dose.g Utilize therapeutic drug monitoring to guide dose adjustments.*Patients may receive an initial low dose of a tricyclic, which is then increased over several days to the recommended steady-state dose. The starting dose in this guideline refers to the recommended steady-state dose. Titrate dose to observed clinical response with symptom improvement and minimal (if any) side effects. Dosing recommendations only apply to higher initial doses of TCAs for treatment of conditions such as depression.*
Guideline strength	Optional
**Drug: fluvoxamine**	
**Diplotype: *3/*4**	
Implications	Greatly reduced metabolism when compared to normal metabolizers. Higher plasma concentrations may increase the probability of side effects.
Metabolizer Status	Poor Metabolizer
Phenotype (Genotype)	An individual carrying only no functional alleles.

**Drug: fluvoxamine**	
**Diplotype: *3/*4**	
Recommendations	Consider a 25-50% reduction of recommended starting dose and titrate to response or use an alternative drug not metabolized by CYP2D6.*Dose extrapolations based on differences in pharmacokinetic parameters between phenotype groups suggest a 30% dose reduction of fluvoxamine. However, a 30% decrease in dose may not be feasible given the dosage forms, therefore, decreasing the starting dose of fluvoxamine by 25-50% should be considered. Drug-drug interactions and other patient characteristics (e.g., age, renal function, liver function) should be considered when selecting an alternative therapy.*
Guideline strength	Optional
**Drug: nortriptyline**	
**Diplotype: *3/*4**	
Implications	Greatly reduced metabolism of TCAs to less active compounds compared to normal metabolizers. Higher plasma concentrations of active drug will increase the probability of side effects.
Metabolizer Status	Poor Metabolizer
Phenotype (Genotype)	An individual carrying only no function alleles.
Recommendations	Avoid tricyclic use due to potential for side effects. Consider alternative drug not metabolized by CYP2D6.If a TCA is warranted, consider 50% reduction of recommended starting dose.g Utilize therapeutic drug monitoring to guide dose adjustments.*Patients may receive an initial low dose of a tricyclic, which is then increased over several days to the recommended steady-state dose. The starting dose in this guideline refers to the recommended steady-state dose. Titrate dose to observed clinical response with symptom improvement and minimal (if any) side effects. Dosing recommendations only apply to higher initial doses of TCAs for treatment of conditions such as depression.*
Guideline strength	Strong
**Drug: ondansetron**	
**Diplotype: *3/*4**	
Implications	Very limited data available for CYP2D6 poor metabolizers.
Metabolizer Status	Poor Metabolizer
Phenotype (Genotype)	An individual carrying no functional alleles.
Recommendations	Insufficient evidence demonstrating clinical impact based on CYP2D6 genotype. Initiate therapy with recommended starting dose.
Guideline strength	No recommendation

**Drug: paroxetine**	
**Diplotype: *3/*4**	
Implications	Greatly reduced metabolism when compared to normal metabolizers. Higher plasma concentrations may increase the probability of side effects.
Metabolizer Status	Poor Metabolizer
Phenotype (Genotype)	An individual carrying only no functional alleles.
Recommendations	Select alternative drug not predominantly metabolized by CYP2D6 or if paroxetine use warranted, consider a 50% reduction of recommended starting dose and titrate to response.*Drug-drug interactions and other patient characteristics (e.g., age, renal function, liver function) should be considered when selecting an alternative therapy.*
Guideline strength	Optional
**Drug: tamoxifen**	
**Diplotype: *3/*4**	
Implications	Lower endoxifen concentrations compared to normal metabolizers; higher risk of breast cancer recurrence, event-free and recurrence-free survival compared to normal metabolizers.
Metabolizer Status	Poor Metabolizer
Phenotype (Genotype)	An individual carrying only no functional alleles.
Recommendations	Recommend alternative hormonal therapy such as an aromatase inhibitor for postmenopausal women or aromatase inhibitor along with ovarian function suppression in premenopausal women given that these approaches are superior to tamoxifen regardless of CYP2D6 genotype [Articles:26211827, 24881463] and based on knowledge that CYP2D6 poor metabolizers switched from tamoxifen to anastrozole do not have an increased risk of recurrence [Article:23213055]. Note, higher dose tamoxifen (40 mg/day) increases but does not normalize endoxifen concentrations and can be considered if there are contraindications to aromatase inhibitor therapy [Articles:27226358, 21768473].
Guideline strength	Strong
**Drug: tropisetron**	
**Diplotype: *3/*4**	
Implications	Very limited data available for CYP2D6 poor metabolizers.
Metabolizer Status	Poor Metabolizer
Phenotype (Genotype)	An individual carrying no functional alleles.
Recommendations	Insufficient evidence demonstrating clinical impact based on CYP2D6 genotype. Initiate therapy with recommended starting dose.
Guideline strength	No recommendation

**Gene(s)**	**CYP3A5**
**All possible diplotype(s)**	***3/*3**
**# missing variants (CYP3A5)**	**0/8**
**Drug: tacrolimus**	
**Diplotype: *3/*3**	
Implications	Higher (“normal”) dose-adjusted trough concentrations of tacrolimus and increased chance of achieving target tacrolimus concentrations.
Metabolizer Status	Poor Metabolizer
Phenotype (Genotype)	An individual carrying two no function alleles (CYP3A5 non-expresser).
Recommendations	Initiate therapy with standard recommended dose. Use therapeutic drug monitoring to guide dose adjustments.
Guideline strength	Strong

**Gene(s)**	**DPYD**
**All possible diplotype(s)**	**No CPIC decreased or no function variant with strong or moderate evidence found**
**# missing variants (DPYD)**	**0/10**
**Drug: capecitabine**	
**Diplotype: No CPIC decreased or no function variant with strong or moderate evidence found**	
Implications	Normal DPD activity and "normal" risk for fluoropyrimidine toxicity.
Metabolizer Status	Normal Metabolizer
Phenotype (Genotype)	An individual carrying two normal function alleles.
Recommendations	Based on genotype, there is no indication to change dose or therapy. Use label-recommended dosage and administration.
Guideline strength	Strong
**Drug: fluorouracil**	
**Diplotype: No CPIC decreased or no function variant with strong or moderate evidence found**	
Implications	Normal DPD activity and "normal" risk for fluoropyrimidine toxicity.
Metabolizer Status	Normal Metabolizer
Phenotype (Genotype)	An individual carrying two normal function alleles.
Recommendations	Based on genotype, there is no indication to change dose or therapy. Use label-recommended dosage and administration.
Guideline strength	Strong

**Gene(s)**	**G6PD**
**All possible diplotype(s)**	**B (wildtype)/B (wildtype)**
**# missing variants (G6PD)**	**0/166**
**Drug: rasburicase**	
**Diplotype: B (wildtype)/B (wildtype)**	
Implications	Low or reduced risk of hemolytic anemia.
Metabolizer Status	N/A
Phenotype (Genotype)	Normal. A female carrying two non-deficient (class IV) alleles, A male carrying a non-deficient (class IV) allele.*Note: A negative or inconclusive genetic test cannot be assumed to indicate normal G6PD phenotype; an enzyme activity test is needed to assign G6PD phenotype in such cases.*
Recommendations	No reason to withhold rasburicase based on G6PD status.
Guideline strength	Strong

**Gene(s)**	**IFNL3**
**All possible diplotype(s)**	**No info**
**# missing variants (IFNL3)**	**1/1**
**Drug: peginterferon-alfa-2a, peginterferon-alfa-2b, ribavirin**	
**Diplotype: No info**	
No Guideline.	

**Gene(s)**	**NUDT15, TPMT**
**All possible diplotype(s)**	**[*1/*1, *1/*1]**
**# missing variants (NUDT15)**	**0/17**
**# missing variants (TPMT)**	**0/30**
**Drug: azathioprine, mercaptopurine**	
**Diplotype: [*1/*1, *1/*1]**	
No Guideline.	

**Gene(s)**	**SLCO1B1**
**All possible diplotype(s)**	**rs4149056C/rs4149056T**
**# missing variants (SLCO1B1)**	**1/29**
**Drug: simvastatin**	
**Diplotype: rs4149056C/rs4149056T**	
Implications	Intermediate myopathy risk
Metabolizer Status	N/A
Phenotype (Genotype)	Decreased Function*CPIC Term Standardization Project recommended replacing the term ‘intermediate’ function with ‘decreased’ function [Article:27441996] for transporters.*
Recommendations	Prescribe a lower dose or consider an alternative statin (e.g., pravastatin or rosuvastatin); consider routine creatine kinase (CK) surveillance
Guideline strength	Strong
**Gene(s)**	**UGT1A1**
**All possible diplotype(s)**	***1/*1**
**# missing variants (UGT1A1)**	**3/5**
**Drug: atazanavir**	
**Diplotype: *1/*1**	
Implications	Reference UGT1A1 activity; very low likelihood of bilirubin-related discontinuation of atazanavir.*“Reference” function refers to the UGT1A1 allele to which other alleles are compared.*
Metabolizer Status	Normal Metabolizer
Phenotype (Genotype)	An individual carrying 2 reference function and/or increased function alleles; or individuals of genotype CC at rs887829*The term 'reference' function refers to the UGT1A1 alleles to which other alleles are compared. The reference function *1 allele is fully functional and refers to the rs8175347 TA6 allele.*
Recommendations	There is no need to avoid prescribing of atazanavir based on *UGT1A1* genetic test result.*Inform the patient that some patients stop atazanavir because of jaundice (yellow eyes and skin), but that this patient’s genotype makes this unlikely (less than about a 1 in 20 chance of stopping atazanavir because of jaundice).*
Guideline strength	Strong

DISCLAIMER:

PharmVIP platform is currently provided for research and informational purposes only and is not intended to diagnose, treat, cure, or prevent any disease. The report generated by the platform is not meant to be a substitute for professional medical advice, diagnosis, or treatment provided by a physician or other qualified health care professional. Only a physician, pharmacist or other healthcare professional should advise a patient on the use of the medications prescribed. Any application of the content provided or obtained through the use of the platform is therefore solely at the user's own risk and responsibility.
