# Supplementary material for: PharmVIP: A Web-Based Tool for Pharmacogenomic Variant Analysis and Interpretation
Source: J Pers Med. 2021 Nov 19;11(11):1230. doi: 10.3390/jpm11111230 (PMC8618518; doi:10.3390/jpm11111230)
Supplement: Supplementary file 1 [file jpm-11-01230-s001.zip › File_S1.pdf]

PharmVIP analysis

Project details

**Project title:**	test
**Analysis date:**	17 August 2021 4:08 p.m.
**Analyzed by:**	Hello World

Options & parameters

**Guideline:**	17 genes (CACNA1S, CFTR, CYP2B6, CYP2C19, CYP2C9, CYP2D6, CYP3A5, CYP4F2, DPYD, G6PD, IFNL3, NUDT15, RYR1, SLCO1B1, TPMT, UGT1A1, VKORC1)	
**HLA:**	All HLA genes, Cohort ethnicity (Caucasian, Diverse, European, Han Chinese, Indian, Japanese, Korean, Thai, Unknown, Vietnamese), p-value < 0.05	
**PGx genes:**	19 genes, (CPIC guideline 2019,) Transcript database (RefSeq transcripts)	
**Input files:**		
**Guideline:**	VCF:	1/1/input/vcf_sample.vcf.gz
	BAM:	1/1/input/bam_sample.bam
**HLA:**	BAM:	1/1/input/bam_sample.bam
**PGx genes:**	VCF:	1/1/input/vcf_sample.vcf.gz

Summary: Guideline

Related CPIC guideline(s)

CACNA1S, RYR1				
**Drug name**	**Diplotype**	**Phenotype**	**Recommendations**	**Guideline strength**
desflurane, enflurane, halothane, isoflurane, methoxyflurane, sevoflurane, succinylcholine	Reference/Reference, Reference/Reference	N/A, N/A	Clinical findings, family history, further genetic testing and other laboratory data should guide use of halogenated volatile anesthetics or depolarizing muscle relaxants.	Strong
**CFTR**				
**Drug name**	**Diplotype**	**Phenotype**	**Recommendations**	**Guideline strength**
ivacaftor	No CPIC variants found	N/A	This guideline does not contain recommendations for this allele combination.	N/A
**CYP2B6**				
**Drug name**	**Diplotype**	**Phenotype**	**Recommendations**	**Guideline strength**
efavirenz	*1/*1	Normal Metabolizer	Initiate efavirenz with standard dosing (600 mg/day).	Strong
**CYP2C19**				
**Drug name**	**Diplotype**	**Phenotype**	**Recommendations**	**Guideline strength**
citalopram, escitalopram	*1/*2	Intermediate Metabolizer	Initiate therapy with recommended starting dose.	Strong
clopidogrel	*1/*2	Intermediate Metabolizer	Alternative antiplatelet therapy (if no contraindication); e.g., prasugrel, ticagrelor	Moderate
sertraline	*1/*2	Intermediate Metabolizer	Initiate therapy with recommended starting dose.	Strong
voriconazole	*1/*2	Intermediate Metabolizer	For pediatric or adult patients: initiate therapy with recommended standard of care dosing.	Moderate

CYP2C19, CYP2D6				
**Drug name**	**Diplotype**	**Phenotype**	**Recommendations**	**Guideline strength**
amitriptyline	*1/*2,<br>*3/*4	Intermediate Metabolizer, Poor Metabolizer	Avoid amitriptyline use. If amitriptyline is warranted, consider 50% reduction of recommended starting dose.	Optional
clomipramine	*1/*2,<br>*3/*4	Intermediate Metabolizer, Poor Metabolizer	Avoid amitriptyline use. If amitriptyline is warranted, consider 50% reduction of recommended starting dose.	Optional
doxepin	*1/*2,<br>*3/*4	Intermediate Metabolizer, Poor Metabolizer	Avoid amitriptyline use. If amitriptyline is warranted, consider 50% reduction of recommended starting dose.	Optional
imipramine	*1/*2,<br>*3/*4	Intermediate Metabolizer, Poor Metabolizer	Avoid amitriptyline use. If amitriptyline is warranted, consider 50% reduction of recommended starting dose.	Optional
trimipramine	*1/*2,<br>*3/*4	Intermediate Metabolizer, Poor Metabolizer	Avoid amitriptyline use. If amitriptyline is warranted, consider 50% reduction of recommended starting dose.	Optional

CYP2C9, CYP4F2, VKORC1				
**Drug name**	**Diplotype**	**Phenotype**	**Recommendations**	**Guideline strength**
warfarin	*1/*2,<br>*1/*1,<br>No info	N/A, N/A, N/A	No Guideline.	N/A

CYP2D6				
**Drug name**	**Diplotype**	**Phenotype**	**Recommendations**	**Guideline strength**
atomoxetine	*3/*4	Poor Metabolizer	**Children:**<br>Initiate with a dose of 0.5 mg/kg/day and if no clinical response and in the absence of adverse events after 2 weeks, consider obtaining a plasma concentration 4 h after dosing. If response is inadequate and concentration is <200 ng/ml, consider a proportional dose increase to achieve a concentration to approach 400 ng/ml. If unacceptable side effects are present at any time, consider a reduction in dose.<br>**Strength of recommendation:**<br>Strong **Adults:**<br>Initiate with a dose of 40 mg/day and if no clinical response and in the absence of adverse events after 2 weeks increase dose to 80 mg/day. If response is inadequate after 2 weeks consider obtaining a plasma concentration 2-4 h after dosing. If concentration is <200 ng/ml, consider a proportional dose increase to achieve a concentration to approach 400 ng/ml. If unacceptable side effects are present at any time, consider a reduction in dose.<br>**Strength of recommendation:**<br>Moderate	Moderate
codeine	*3/*4	Poor Metabolizer	Avoid codeine use due to lack of efficacy.	Strong

CYP2D6				
Drug name	Diplotype	Phenotype	Recommendations	Guideline strength
desipramine	*3/*4	Poor Metabolizer	Avoid tricyclic use due to potential for side effects. Consider alternative drug not metabolized by CYP2D6. If a TCA is warranted, consider 50% reduction of recommended starting dose.g Utilize therapeutic drug monitoring to guide dose adjustments.	Optional
fluvoxamine	*3/*4	Poor Metabolizer	Consider a 25-50% reduction of recommended starting dose and titrate to response or use an alternative drug not metabolized by CYP2D6.	Optional
nortriptyline	*3/*4	Poor Metabolizer	Avoid tricyclic use due to potential for side effects. Consider alternative drug not metabolized by CYP2D6. If a TCA is warranted, consider 50% reduction of recommended starting dose.g Utilize therapeutic drug monitoring to guide dose adjustments.	Strong
ondansetron	*3/*4	Poor Metabolizer	Insufficient evidence demonstrating clinical impact based on CYP2D6 genotype. Initiate therapy with recommended starting dose.	No recommendation
paroxetine	*3/*4	Poor Metabolizer	Select alternative drug not predominantly metabolized by CYP2D6 or if paroxetine use warranted, consider a 50% reduction of recommended starting dose and titrate to response.	Optional

CYP2D6				
**Drug name**	**Diplotype**	**Phenotype**	**Recommendations**	**Guideline strength**
tamoxifen	*3/*4	Poor Metabolizer	Recommend alternative hormonal therapy such as an aromatase inhibitor for postmenopausal women or aromatase inhibitor along with ovarian function suppression in premenopausal women given that these approaches are superior to tamoxifen regardless of CYP2D6 genotype [Articles:26211827, 24881463] and based on knowledge that CYP2D6 poor metabolizers switched from tamoxifen to anastrozole do not have an increased risk of recurrence [Article:23213055]. Note, higher dose tamoxifen (40 mg/day) increases but does not normalize endoxifen concentrations and can be considered if there are contraindications to aromatase inhibitor therapy [Articles:27226358, 21768473].	Strong
tropisetron	*3/*4	Poor Metabolizer	Insufficient evidence demonstrating clinical impact based on CYP2D6 genotype. Initiate therapy with recommended starting dose.	No recommendation

CYP3A5				
**Drug name**	**Diplotype**	**Phenotype**	**Recommendations**	**Guideline strength**
tacrolimus	*3/*3	Poor Metabolizer	Initiate therapy with standard recommended dose. Use therapeutic drug monitoring to guide dose adjustments.	Strong

CONTACT
National Biobank of Thailand (NBT)
Tel : 025647000 Ext. 71475
Email : nbt@nstda.or.th

DPYD				
**Drug name**	**Diplotype**	**Phenotype**	**Recommendations**	**Guideline strength**
capecitabine	No CPIC decreased or no function variant with strong or moderate evidence found	Normal Metabolizer	Based on genotype, there is no indication to change dose or therapy. Use label-recommended dosage and administration.	Strong
fluorouracil	No CPIC decreased or no function variant with strong or moderate evidence found	Normal Metabolizer	Based on genotype, there is no indication to change dose or therapy. Use label-recommended dosage and administration.	Strong

G6PD				
**Drug name**	**Diplotype**	**Phenotype**	**Recommendations**	**Guideline strength**
rasburicase	B (wildtype)/B (wildtype)	N/A	No reason to withhold rasburicase based on G6PD status.	Strong

IFNL3				
**Drug name**	**Diplotype**	**Phenotype**	**Recommendations**	**Guideline strength**
peginterferon-alfa-2a, peginterferon-alfa-2b, ribavirin	No info	N/A	No Guideline.	N/A

NUDT15, TPMT				
**Drug name**	**Diplotype**	**Phenotype**	**Recommendations**	**Guideline strength**
azathioprine, mercaptopurine	*1/*1, *1/*1	N/A, N/A	No Guideline.	N/A

SLCO1B1				
**Drug name**	**Diplotype**	**Phenotype**	**Recommendations**	**Guideline strength**
simvastatin	rs4149056C/rs4149056T	N/A	Prescribe a lower dose or consider an alternative statin (e.g., pravastatin or rosuvastatin); consider routine creatine kinase (CK) surveillance	Strong

UGT1A1				
**Drug name**	**Diplotype**	**Phenotype**	**Recommendations**	**Guideline strength**
atazanavir	*1/*1	Normal Metabolizer	There is no need to avoid prescribing of atazanavir based on *UGT1A1* genetic test result.	Strong

CONTACT
National Biobank of Thailand (NBT)
Tel : 025647000 Ext. 71475
Email : nbt@nstda.or.th

Summary: HLA

Related CPIC guideline(s)

CYP2C9, HLA-B			
**Drug name**	**Diplotype**	**Recommendations**	**Guideline strength**
phenytoin	*1/*2, Other/Other	Consider 25% reduction of recommended starting maintenance dosed (based on patient's clinical characteristics). Subsequent maintenance doses should be adjusted according to therapeutic drug monitoring and response.	Moderate

HLA-A, HLA-B			
**Drug name**	**Diplotype**	**Recommendations**	**Guideline strength**
carbamazepine	Other/Other, Other/Other	Use carbamazepine per standard dosing guidelines	Strong

HLA-B			
**Drug name**	**Diplotype**	**Recommendations**	**Guideline strength**
abacavir	Other/Other	Use abacavir per standard dosing guidelines	Strong
allopurinol	Other/Other	Use allopurinol per standard dosing guidelines.	Strong
oxcarbazepine	Other/Other	Use oxcarbazepine per standard dosing guidelines	Strong

Related publication(s) for HLA-ADR association

HLA-A*01 (No. of detecting tools = 3/3)				
**Drug name**	**ADR**	**Patient disease**	**Cohort ethnicity**	**No. of pub.(s)**
Clozapine	agranulocytosis	Schizophrenia	Caucasian	1

HLA-A*01:01 (No. of detecting tools = 3/3)				
**Drug name**	**ADR**	**Patient disease**	**Cohort ethnicity**	**No. of pub.(s)**
Amoxicillin-clavulanate	drug induced liver injury (DILI)	Bacterial infections	Caucasian	2
Antibacterial drugs	Stevens–Johnson syndrome (SJS),toxic epidermal necrolysis (TEN),drug-induced hypersensitivity syndrome (DIHS),acute generalized exanthematous pustulosis (AGEP)	Bacterial infections	Unknown	2
Antiepileptic drugs	Stevens-Johnson syndrome (SJS),toxic epidermal necrolysis (TEN)	Epilepsy	Han Chinese	1
Antiepileptic drugs	Stevens–Johnson syndrome (SJS),toxic epidermal necrolysis (TEN),drug-induced hypersensitivity syndrome (DIHS),acute generalized exanthematous pustulosis (AGEP)	Epilepsy	Unknown	1
Carbamazepine	Stevens-Johnson syndrome (SJS),toxic epidermal necrolysis (TEN),drug-induced hypersensitivity syndrome (DIHS)	Epilepsy	Japanese	1
Carbamazepine	Stevens-Johnson syndrome (SJS),toxic epidermal necrolysis (TEN)	Epilepsy	Han Chinese	2
Carbamazepine	Stevens-Johnson syndrome (SJS),toxic epidermal necrolysis (TEN)	Epilepsy	European	2
EGF receptor inhibitors	maculopapular exanthema (MPE)	Cancer	Unknown	1
Lamotrigine	Stevens–Johnson syndrome (SJS),toxic epidermal necrolysis (TEN)	Epilepsy	Caucasian	1
Lamotrigine	drug-induced hypersensitivity syndrome (DIHS)	Epilepsy	Caucasian	1
Levetiracetam	maculopapular eruption (MPE)	Epilepsy	Indian	1
NSAID and 'multi-ingredient cold medication'	Stevens-Johnson syndrome (SJS),toxic epidermal necrolysis (TEN),severe occular complicatoins	Common cold	Diverse	1

HLA-A*11 (No. of detecting tools = 3/3)				
**Drug name**	**ADR**	**Patient disease**	**Cohort ethnicity**	**No. of pub.(s)**
Tetanus antitoxin	maculopapular eruption (MPE)	Tetanus	Han Chinese	1

CONTACT
National Biobank of Thailand (NBT)
Tel : 025647000 Ext. 71475
Email : nbt@nstda.or.th

HLA-A*11:01 (No. of detecting tools = 2/3)				
**Drug name**	**ADR**	**Patient disease**	**Cohort ethnicity**	**No. of pub.(s)**
Amoxicillin-clavulanate	drug induced liver injury (DILI)	Bacterial infections	Caucasian	2
Antibacterial drugs	Stevens–Johnson syndrome (SJS),toxic epidermal necrolysis (TEN),drug-induced hypersensitivity syndrome (DIHS),acute generalized exanthematous pustulosis (AGEP)	Bacterial infections	Unknown	2
Antiepileptic drugs	Stevens–Johnson syndrome (SJS),toxic epidermal necrolysis (TEN),drug-induced hypersensitivity syndrome (DIHS),acute generalized exanthematous pustulosis (AGEP)	Epilepsy	Unknown	1
Carbamazepine	Stevens–Johnson syndrome (SJS),toxic epidermal necrolysis (TEN)	Epilepsy	Caucasian	1
Carbamazepine	Stevens-Johnson syndrome (SJS),toxic epidermal necrolysis (TEN),drug-induced hypersensitivity syndrome (DIHS)	Epilepsy	Japanese	1
Carbamazepine	Stevens-Johnson syndrome (SJS),toxic epidermal necrolysis (TEN)	Epilepsy	Han Chinese	4
Carbamazepine	drug-induced hypersensitivity syndrome (DIHS)	Epilepsy	Han Chinese	2
Carbamazepine	maculopapular exanthema (MPE)	Epilepsy	Han Chinese	3
Clarithromycin	drug-induced skin reaction (DISI)	Bacterial infections	Han Chinese	1
Lamotrigine	maculopapular exanthema (MPE)	Epilepsy	Han Chinese	1
Lamotrigine	Stevens–Johnson syndrome (SJS),toxic epidermal necrolysis (TEN)	Epilepsy	Caucasian	1
Levetiracetam	maculopapular eruption (MPE)	Epilepsy	Indian	1
NSAID and 'multi-ingredient cold medication'	Stevens-Johnson syndrome (SJS),toxic epidermal necrolysis (TEN),severe occular complicatoins	Common cold	Diverse	1
Phenytoin	Stevens–Johnson syndrome (SJS),toxic epidermal necrolysis (TEN)	Epilepsy	Caucasian	1
Phenytoin	Stevens-Johnson syndrome (SJS),toxic epidermal necrolysis (TEN)	Epilepsy	Han Chinese	1
Tetanus antitoxin	maculopapular eruption (MPE)	Tetanus	Han Chinese	1

HLA-B*08:01 (No. of detecting tools = 3/3)				
**Drug name**	**ADR**	**Patient disease**	**Cohort ethnicity**	**No. of pub.(s)**
Amoxicillin-clavulanate	drug induced liver injury (DILI)	Bacterial infections	Caucasian	2
Antibacterial drugs	Stevens–Johnson syndrome (SJS),toxic epidermal necrolysis (TEN),drug-induced hypersensitivity syndrome (DIHS),acute generalized exanthematous pustulosis (AGEP)	Bacterial infections	Unknown	2
Antiepileptic drugs	Stevens–Johnson syndrome (SJS),toxic epidermal necrolysis (TEN),drug-induced hypersensitivity syndrome (DIHS),acute generalized exanthematous pustulosis (AGEP)	Epilepsy	Unknown	1
Carbamazepine	Stevens-Johnson syndrome (SJS),toxic epidermal necrolysis (TEN)	Epilepsy	European	2
Carbamazepine	Stevens-Johnson syndrome (SJS),toxic epidermal necrolysis (TEN),drug-induced hypersensitivity syndrome (DIHS)	Epilepsy	Vietnamese	1
EGF receptor inhibitors	maculopapular exanthema (MPE)	Cancer	Unknown	1
Lamotrigine	drug-induced hypersensitivity syndrome (DIHS)	Epilepsy	Caucasian	1
NSAID and 'multi-ingredient cold medication'	Stevens-Johnson syndrome (SJS),toxic epidermal necrolysis (TEN),severe occular complicatoins	Common cold	Diverse	1
Phenytoin	Stevens–Johnson syndrome (SJS),toxic epidermal necrolysis (TEN)	Epilepsy	Caucasian	1

HLA-B*56:01 (No. of detecting tools = 3/3)				
**Drug name**	**ADR**	**Patient disease**	**Cohort ethnicity**	**No. of pub.(s)**
Amoxicillin-clavulanate	drug induced liver injury (DILI)	Bacterial infections	Caucasian	2
Antibacterial drugs	Stevens–Johnson syndrome (SJS),toxic epidermal necrolysis (TEN),drug-induced hypersensitivity syndrome (DIHS),acute generalized exanthematous pustulosis (AGEP)	Bacterial infections	Unknown	2
Antiepileptic drugs	Stevens–Johnson syndrome (SJS),toxic epidermal necrolysis (TEN),drug-induced hypersensitivity syndrome (DIHS),acute generalized exanthematous pustulosis (AGEP)	Epilepsy	Unknown	1
Carbamazepine	Stevens-Johnson syndrome (SJS),toxic epidermal necrolysis (TEN)	Epilepsy	Thai	1

HLA-B*56:01 (No. of detecting tools = 3/3)				
**Drug name**	**ADR**	**Patient disease**	**Cohort ethnicity**	**No. of pub.(s)**
Carbamazepine	Stevens-Johnson syndrome (SJS),toxic epidermal necrolysis (TEN),drug-induced hypersensitivity syndrome (DIHS),maculopapular exanthema (MPE)	Epilepsy	Japanese	1
Carbamazepine	Stevens-Johnson syndrome (SJS),toxic epidermal necrolysis (TEN)	Epilepsy	Han Chinese	1
NSAID and 'multi-ingredient cold medication'	Stevens-Johnson syndrome (SJS),toxic epidermal necrolysis (TEN),severe occular complicatoins	Common cold	Diverse	1
Oxcarbazepine	maculopapular exanthema (MPE)	Epilepsy	Han Chinese	1
Phenytoin	Stevens-Johnson syndrome (SJS),toxic epidermal necrolysis (TEN)	Epilepsy	Han Chinese	1

HLA-C*01:02 (No. of detecting tools = 3/3)				
**Drug name**	**ADR**	**Patient disease**	**Cohort ethnicity**	**No. of pub.(s)**
Carbamazepine	drug-induced hypersensitivity syndrome (DIHS)	Epilepsy	Han Chinese	1
Carbamazepine	maculopapular exanthema (MPE)	Epilepsy	Han Chinese	1
Carbamazepine	Stevens-Johnson syndrome (SJS)	Epilepsy	Korean	1
Carbamazepine	drug-induced hypersensitivity syndrome (DIHS)	Epilepsy	Korean	1
Carbamazepine	drug-induced skin reaction (DISI)	Epilepsy	Korean	1
Carbamazepine	Stevens-Johnson syndrome (SJS),toxic epidermal necrolysis (TEN)	Epilepsy	Han Chinese	1
Clarithromycin	drug-induced skin reaction (DISI)	Bacterial infections	Han Chinese	1
Lamotrigine	maculopapular eruption (MPE)	Epilepsy	Korean	1
NSAID and 'multi-ingredient cold medication'	Stevens-Johnson syndrome (SJS),toxic epidermal necrolysis (TEN),severe occular complicatoins	Common cold	Diverse	1
Phenytoin	Stevens-Johnson syndrome (SJS),toxic epidermal necrolysis (TEN)	Epilepsy	Han Chinese	1
Tetanus antitoxin	maculopapular eruption (MPE)	Tetanus	Han Chinese	1

HLA-C*07 (No. of detecting tools = 3/3)				
**Drug name**	**ADR**	**Patient disease**	**Cohort ethnicity**	**No. of pub.(s)**
Clozapine	agranulocytosis	Schizophrenia	Caucasian	1

HLA-C*07:01 (No. of detecting tools = 3/3)				
**Drug name**	**ADR**	**Patient disease**	**Cohort ethnicity**	**No. of pub.(s)**
EGF receptor inhibitors	maculopapular exanthema (MPE)	Cancer	Unknown	1
Lamotrigine	Stevens–Johnson syndrome (SJS),toxic epidermal necrolysis (TEN)	Epilepsy	Caucasian	1
Lamotrigine	drug-induced hypersensitivity syndrome (DIHS)	Epilepsy	Caucasian	1
Lamotrigine	maculopapular eruption (MPE)	Epilepsy	Korean	1
NSAID and 'multi-ingredient cold medication'	Stevens-Johnson syndrome (SJS),toxic epidermal necrolysis (TEN),severe occular complicatoins	Common cold	Diverse	1
Phenytoin	Stevens–Johnson syndrome (SJS),toxic epidermal necrolysis (TEN)	Epilepsy	Caucasian	1
Phenytoin	drug-induced hypersensitivity syndrome (DIHS)	Epilepsy	Caucasian	1

HLA-DPB1*04:01 (No. of detecting tools = 3/3)				
**Drug name**	**ADR**	**Patient disease**	**Cohort ethnicity**	**No. of pub.(s)**
Bucillamine	bucillamine-induced proteinuria	Rheumatoid arthritis	Japanese	1

HLA-DPB1*14:01 (No. of detecting tools = 3/3)				
**Drug name**	**ADR**	**Patient disease**	**Cohort ethnicity**	**No. of pub.(s)**
Bucillamine	bucillamine-induced proteinuria	Rheumatoid arthritis	Japanese	1

HLA-DQA1*01:01 (No. of detecting tools = 3/3)				
**Drug name**	**ADR**	**Patient disease**	**Cohort ethnicity**	**No. of pub.(s)**
Amoxicillin-clavulanate	drug induced liver injury (DILI)	Bacterial infections	Caucasian	2

HLA-DQA1*05:01 (No. of detecting tools = 3/3)				
**Drug name**	**ADR**	**Patient disease**	**Cohort ethnicity**	**No. of pub.(s)**
Amoxicillin-clavulanate	drug induced liver injury (DILI)	Bacterial infections	Caucasian	2

HLA-DQB1*02:01 (No. of detecting tools = 3/3)				
**Drug name**	**ADR**	**Patient disease**	**Cohort ethnicity**	**No. of pub.(s)**
Amoxicillin-clavulanate	drug induced liver injury (DILI)	Bacterial infections	Caucasian	2
Clozapine	agranulocytosis	Schizophrenia	Caucasian	1

HLA-DQB1*05:01 (No. of detecting tools = 3/3)				
**Drug name**	**ADR**	**Patient disease**	**Cohort ethnicity**	**No. of pub.(s)**
Amoxicillin-clavulanate	drug induced liver injury (DILI)	Bacterial infections	Caucasian	2
Bucillamine	bucillamine-induced proteinuria	Rheumatoid arthritis	Japanese	1
Oxcarbazepine	maculopapular exanthema (MPE)	Epilepsy	Korean	1

HLA-DRB1*01 (No. of detecting tools = 3/3)				
**Drug name**	**ADR**	**Patient disease**	**Cohort ethnicity**	**No. of pub.(s)**
Flucloxacillin	drug induced liver injury (DILI)	Bacterial infections	Caucasian	1

HLA-DRB1*01:01 (No. of detecting tools = 3/3)				
**Drug name**	**ADR**	**Patient disease**	**Cohort ethnicity**	**No. of pub.(s)**
Amoxicillin-clavulanate	drug induced liver injury (DILI)	Bacterial infections	Caucasian	2
Antiepileptic drugs	Stevens-Johnson syndrome (SJS),toxic epidermal necrolysis (TEN)	Epilepsy	Han Chinese	1
Bucillamine	bucillamine-induced proteinuria	Rheumatoid arthritis	Japanese	1
Carbamazepine	maculopapular exanthema (MPE)	Epilepsy	Han Chinese	1
Carbamazepine	Stevens-Johnson syndrome (SJS),toxic epidermal necrolysis (TEN)	Epilepsy	Han Chinese	1
Clozapine	agranulocytosis	Schizophrenia	Caucasian	1
Co-trimoxazole	maculopapular exanthema (MPE),Stevens-Johnson syndrome (SJS)	HIV	Caucasian	1
EGF receptor inhibitors	maculopapular exanthema (MPE)	Cancer	Unknown	1
Nevirapine	drug-induced hypersensitivity syndrome (DIHS)	HIV	Diverse	1
Nevirapine	drug-induced skin reaction (DISI),drug induced liver injury (DILI)	HIV	Caucasian	1

HLA-DRB1*03 (No. of detecting tools = 3/3)				
**Drug name**	**ADR**	**Patient disease**	**Cohort ethnicity**	**No. of pub.(s)**
Flucloxacillin	drug induced liver injury (DILI)	Bacterial infections	Caucasian	1

HLA-DRB1*03:01 (No. of detecting tools = 3/3)				
**Drug name**	**ADR**	**Patient disease**	**Cohort ethnicity**	**No. of pub.(s)**
Allopurinol	Stevens-Johnson syndrome (SJS),toxic epidermal necrolysis (TEN),drug-induced hypersensitivity syndrome (DIHS)	Gout	Han Chinese	1
Amoxicillin-clavulanate	drug induced liver injury (DILI)	Bacterial infections	Caucasian	2
Carbamazepine	maculopapular exanthema (MPE)	Epilepsy	Han Chinese	1
Clozapine	agranulocytosis	Schizophrenia	Caucasian	1
Co-trimoxazole	maculopapular exanthema (MPE),Stevens-Johnson syndrome (SJS)	HIV	Caucasian	1
Nevirapine	drug-induced hypersensitivity syndrome (DIHS)	HIV	Diverse	1
Phenytoin	Stevens-Johnson syndrome (SJS),toxic epidermal necrolysis (TEN)	Epilepsy	Han Chinese	1

HLA-DRB3*01:01 (No. of detecting tools = 3/3)				
**Drug name**	**ADR**	**Patient disease**	**Cohort ethnicity**	**No. of pub.(s)**
Clozapine	agranulocytosis	Schizophrenia	Caucasian	1

Summary: PGx genes

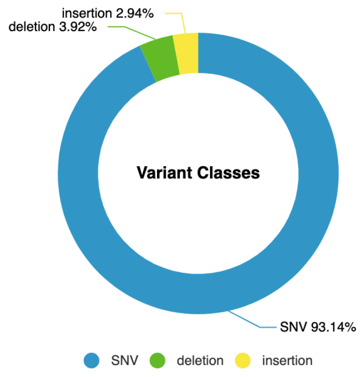

Number of variants categorized by classes	
**Variant classes**	**# variants**
**SNV**	95
**deletion**	4
**insertion**	3

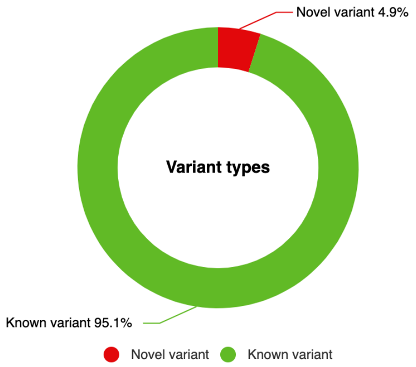

Number of variants categorized by types	
**Type**	**# variants**
**Novel variation**	5
**Known variant**	97

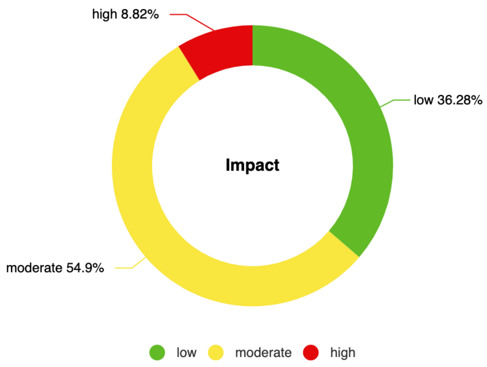

Number of transcripts categorized by variant impacts	
**Impacts**	**# transcripts**
**low**	37
**moderate**	56
**high**	9

CONTACT
National Biobank of Thailand (NBT)
Tel : 025647000 Ext. 71475
Email : nbt@nstda.or.th

Summary report

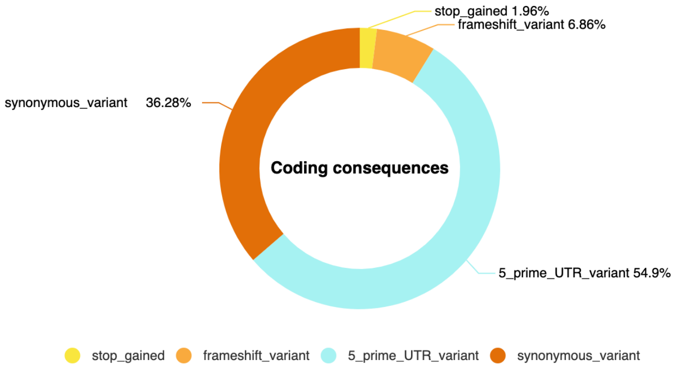

Number of transcripts categorized by coding consequences	
**Coding consequences**	**# transcripts**
**Stop gained**	2
**Frameshift variant**	7
**Missense variant**	56
**Splice region variant**	5
**Synonymous variant**	37

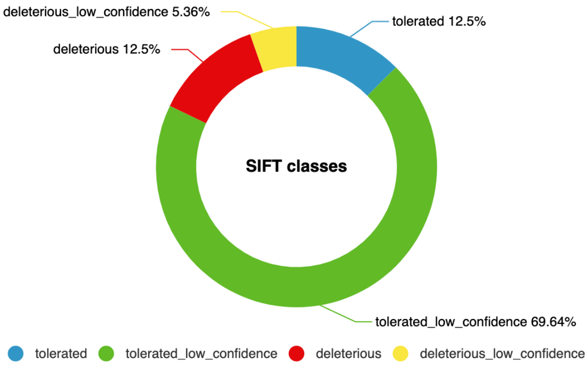

Number of transcripts categorized by SIFT classes	
**SIFT classes**	**# transcripts**
**tolerated**	7
**tolerated_low_confidence**	39
**deleterious**	7
**deleterious_low_confidence**	3

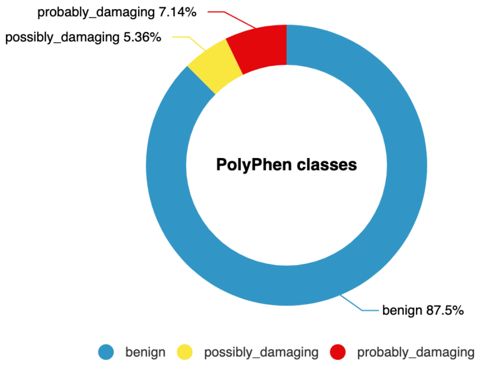

Number of transcripts categorized by PolyPhen classes	
**PolyPhen classes**	**# transcripts**
**benign**	49
**possibly_damaging**	3
**probably_damaging**	4

CONTACT

National Biobank of Thailand (NBT)

Tel : 025647000 Ext. 71475

Email : nbt@nstda.or.th

DISCLAIMER:

PharmVIP platform is currently provided for research and informational purposes only and is not intended to diagnose, treat, cure, or prevent any disease. The report generated by the platform is not meant to be a substitute for professional medical advice, diagnosis, or treatment provided by a physician or other qualified health care professional. Only a physician, pharmacist or other healthcare professional should advise a patient on the use of the medications prescribed. Any application of the content provided or obtained through the use of the platform is therefore solely at the user's own risk and responsibility.
