# Supplementary material for: PharmVIP: A Web-Based Tool for Pharmacogenomic Variant Analysis and Interpretation
Source: J Pers Med. 2021 Nov 19;11(11):1230. doi: 10.3390/jpm11111230 (PMC8618518; doi:10.3390/jpm11111230)
Supplement: Supplementary file 1 [file jpm-11-01230-s001.zip › File_S6.pdf]

The details of discordant diplotypes between PharmVIP and the GeT-RM consensus

We investigated the discordant diplotypes between PharmVIP and the GeT-RM consensus manually to understand the reasons for the discrepancies. In some cases, samples with a consensus diplotype reported by GeT-RM are reported by PharmVIP as an unknown diplotype, e.g. in *CYP3A5* one sample has a haplotype matched to *3, while the other haplotype was composed of both *3 variant and *5 variant on the same haplotype. This sample has a GeT-RM consensus diplotype of *3/*3. In contrast, it is labeled as ?/? by PharmVIP since there is no known allele with both *3 variant and *5 variant present on the same haplotype [1]. Similar scenarios were observed in three samples called by PharmVIP with unknown allele in *CYP4F2*. The possible explanation for discrepancy in diplotype assignment is that the assay platforms may depend on different sets of variant positions (compared with CPIC allele definition table) for defining the alleles and information from these positions may not be able to differentiate the novel alleles from the known alleles. We also explored the other five discordant samples with known alleles. One sample was discordant due to the fact that the *TPMT* predicted allele was not on the list of interrogated alleles by the PGx testing platforms. A sample with discordant *CYP2C19* diplotype showed no consensus, in which three testing platforms and PharmVIP gave all different diplotypes. Another unmatched sample displayed highly similar *CYP2C19* diplotypes between PharmVIP and the GeT-RM consensus (*1/*4A vs *1/*4). The *4A allele was only tested by one out of six platforms, and thus was not displayed as a consensus. For the other two discordant samples in *CYP4F2*, different diplotypes from two testing platforms were obtained, with one diplotype assigned as consensus and the other matched with PharmVIP.

For *CYP2D6*, only 35 out of 88 samples (39.77%) were concordant between PharmVIP and GeT-RM. The predicted diplotypes of 22 out of 53 discordant samples were associated with allele(s) that were not interrogated by any GeT-RM assay platform. For example, the PharmVIP-called diplotype *43/*45 was reported by GeT-RM as consensus *1/*2. The discordance was due to the fact that no PGx testing platform in GeT-RM can detect the *43 and *45 alleles. Investigation of the other three samples showed that the PharmVIP predicted diplotypes were called by more than one assay platform, but the GeT-RM consensus diplotype differed. The discordance of the remaining 28 samples was possibly caused by different allele definitions, such as the different combinations of variants for defining the haplotypes used by the GeT-RM assays, and by the Astrolabe tool employed by PharmVIP and PharmCAT.

For *DPYD*, different allele nomenclatures were used for diplotype calling by genotyping platforms in GeT-RM and the PharmVIP tool. The allele definition table of *DPYD* employed by PharmVIP contains a list of variants such as c.1905+1G>A, c.1129-5923C>G, of which the combinations of these variants are used for determining the dosing recommendations for 5-fluorouracil and capecitabine [2]. On the other hand, the genotyping platforms in GeT-RM are based on the *DPYD* star alleles. The 53.41% concordant samples are all the wild-type diplotype (*1/*1 from GeT-RM and Reference/Reference from PharmVIP) (File S5), while the discordant samples arise from different allele nomenclatures as described above.

SLCO1B1 showed the lowest concordance (22.73%) between PharmVIP and GeT-RM of all pharmacogenes reported (Table 1, File S5). For this gene, the additional rule of allele assignment implemented in PharmCAT was also employed in PharmVIP. PharmVIP performs haplotype-based allele matching for *SLCO1B1* based on star alleles present in the PharmVIP allele definition table in combination with the rs4149056 variant. If sample genotypic data is not matched with any star allele, only the rs4149056 genotype will be reported. For simvastatin, the evidence linking the association between this SNP (rs4149056T>C) and myopathy was reported, whether it is detected as single variant or in combination with other variants. The therapeutic recommendations for simvastatin based on rs4149056 genotype were also provided [3]. Most of the PharmVIP discordant cases (66 of 68 samples) were not matched with star alleles and only the predictions based on rs4149056 were reported. In contrast, GeT-RM reported a star allele consensus. The discordant events could be caused by the fact that the different sets of analyzed variants were considered in each genotyping platform and PharmVIP allele definition table. Upon closer examination, we identified a discordant sample (NA07019) of which the consensus is *1/*14 (*1A/*14 were identified in 2 out of 3 genotyping platforms). PharmVIP could not identify the matched diplotypes in the allele definition table, indicating a possible novel allele. However, without considering 3 out of 29 variant positions for allele matching, the PharmVIP predicted result will be *1A/*14. This points to the effect of choice of variants included for analysis to the result outputs. The same reason could be applied for another sample (NA12813), of which the matched star alleles (*19/*21) were identified from the allele definition table but were discordant with the GeT-RM consensus since an allele (*19) was not tested in any GeT-RM genotyping platform.

For *UGT1A1*, we employed the exception rule of allele assignment implemented in PharmCAT, of which the different allele assignment methods are performed for phased and unphased input data. Since the VCF data analysed were not phased, the predicted diplotypes were based on the unphased method. From the *UGT1A1* allele definition table, each star allele is defined by a single variant. The presence of combinations of *UGT1A* variants on the same haplotype is possible. For unphased output, PharmVIP reports the list of all identified star alleles without phased information. The *80 allele is in very high linkage disequilibrium with *28 and *37 [4]; thus, it is reported as *80+*28 and *80+*37 in PharmVIP. However, the *80 allele was not reported in the GeT-RM results. Therefore, *80 was disregarded in the PharmVIP results for comparison with the GeT-RM consensus. Most of *UGT1A1* unmatched samples (20 of 22 samples) were caused by ambiguity of diplotype called by PharmVIP owing to unphased data. For example, the PharmVIP result showed [*28 (heterozygous), *60 (heterozygous)], which means there is one *28 and one *60 allele. Hence, with unphased data there will be two possible diplotypes, i.e., *60/*28 or *1/*60+*28. In constrast, GeT-RM reported only *60/*28.

References:

1. PharmVar CYP3A5 allele definition Available online:

https://www.pharmvar.org/gene/CYP3A5.

2. Amstutz, U.; Henricks, L.M.; Offer, S.M.; Barbarino, J.; Schellens, J.H.M.; Swen, J.J.; Klein, T.E.; McLeod, H.L.; Caudle, K.E.; Diasio, R.B.; et al. Clinical Pharmacogenetics Implementation Consortium (CPIC) Guideline for Dihydropyrimidine Dehydrogenase Genotype and Fluoropyrimidine Dosing: 2017 Update. *Clin. Pharmacol. Ther.* **2018**, *103*, 210–216, doi:10.1002/cpt.911.

3. Ramsey, L.B.; Johnson, S.G.; Caudle, K.E.; Haidar, C.E.; Voora, D.; Wilke, R.A.; Maxwell, W.D.; McLeod, H.L.; Krauss, R.M.; Roden, D.M.; et al. The Clinical Pharmacogenetics Implementation Consortium Guideline for SLCO1B1 and Simvastatin-Induced Myopathy: 2014 Update. *Clin. Pharmacol. Ther.* 2014, *96*, 423–428.

4. CPIC Guideline for Atazanavir and UGT1A1 Available online: https://cpicpgx.org/guidelines/guideline-for-atazanavir-and-ugt1a1/.
