# Supplementary material for: PharmVIP: A Web-Based Tool for Pharmacogenomic Variant Analysis and Interpretation
Source: J Pers Med. 2021 Nov 19;11(11):1230. doi: 10.3390/jpm11111230 (PMC8618518; doi:10.3390/jpm11111230)
Supplement: Supplementary file 1 [file jpm-11-01230-s001.zip › File_S7.pdf]

Input data preprocessing step of the PharmVIP Guideline module

#CHROM	POS	ID	REF	ALT	QUAL	FILTER	INFO	FORMAT	NA12813
chr1	97078203	.	C	.	.	.	AN=2;DP=40	GT:DP:RGQ	0/0:40:99
chr1	97078204	.	A	.	.	.	AN=2;DP=40	GT:DP:RGQ	0/0:40:99
chr1	97078205	.	C	.	.	.	AN=2;DP=40	GT:DP:RGQ	0/0:40:99
chr1	97078206	.	A	.	.	.	AN=2;DP=40	GT:DP:RGQ	0/0:40:99
chr1	97078207	.	G	.	.	.	AN=2;DP=40	GT:DP:RGQ	0/0:40:99
chr1	97078208	.	C	T	47497.32	.	AN=2;DP=48	GT:AD:DP:GQ:PL	0/1:19,29:48:99:844,0,491
chr1	97078209	.	A	.	.	.	AN=2;DP=40	GT:DP:RGQ	0/0:40:99
chr1	97078210	.	A	.	.	.	AN=2;DP=40	GT:DP:RGQ	0/0:40:99
chr1	97078211	.	A	.	.	.	AN=2;DP=40	GT:DP:RGQ	0/0:40:99
chr1	97078212	.	A	.	.	.	AN=2;DP=40	GT:DP:RGQ	0/0:40:99
chr1	97078213	.	G	.	.	.	AN=2;DP=40	GT:DP:RGQ	0/0:40:99

Here is an example of the required GCVF file for the Guideline module. Rows represent the information of each variant position. According to the allele definition table, the sample genotypic data of the required haplotype positions (check column “#CHROM” and “POS” matched with allele definition table) will be parsed from the VCF file to the input file used for the next allele matching step. The required sample genotypic data includes the variant position in the reference genome (#CHROM and POS), reference allele (REF), alternate non-reference allele (ALT), and genotype data (last column labeled as sample name NA12813). For the genotype data, allele and phase information are provided in the VCF file. Phased positions are labeled with a ‘|’ symbol, while the unphased positions are labeled with a ‘/’ symbol. 0 and 1 represent the reference allele and the alternate non-reference allele, respectively. Consider the example of the two positions 97078203 and 97078208 on chromosome 1 that are required for haplotype analysis according to the allele definition table. The position 97078203 has genotype 0/0 which will be parsed as C/C and labeled as a phased position due to the homozygous alleles. The position 97078208 has genotype 0/1, which will be parsed as C/T and labeled as an unphased position due to the heterozygous alleles. The gene phasing status is determined as “unphased-gene” since at least one unphased-variant exists.

Allele matching step of the PharmVIP Guideline module

The steps for allele matching are as follows:
1) The sample genotypic data obtained from the previous data preprocessing step are converted into regular expression(s) based on the variant phase status of a pharmacogene. A regular expression is represented as a string of bases (genotype data) from all variant positions. Missing variants will be treated as representing any base (‘.’ in regular expression).
2) For a phased gene:
    2.1) Two regular expressions, one for each phased haplotype, are generated.
    2.2) Each pattern of the regular expression is compared with the entries from the allele definition table. Two matched alleles, one for each regular expression, are identified.
    2.3) A diplotype is identified from two matched alleles.

2.4) If there is no matched allele for a regular expression, such regular expression (haplotype) will be shown as unknown (?). For example, if one regular expression is matched with *5 but another regular expression is not matched with any allele, the diplotype is reported as *5/?. If there is no matched allele for both regular expressions, the diplotype is reported as ?/?.

3) For an unphased gene:

3.1) One or two regular expressions will be generated. If there are only unphased variants in an unphased gene, only one regular expression is generated, representing all possibilities of both haplotypes. If some phased variants are present in an unphased gene, two regular expressions are generated.

3.2) Each pattern of the regular expression is compared with the entries from the allele definition table. For each regular expression, more than one matched alleles can be identified if there are unphased variant positions that are heterozygous. Either reference or alternative base can be present on such positions.

3.3) If there is no matched allele for at least one regular expression, the diplotype will be shown as unknown (?/?).

3.4) If there is matched allele(s) for all regular expression(s), all possible diplotypes are listed from the pairing of all matched alleles.

3.5) From all possible diplotypes, the diplotype candidates are identified as the ones that can be matched with sample genotypic data.

4) If there is more than one diplotype candidates and a user selects the best candidate option, the best diplotype candidate(s) will be identified using the best allele selection approach from the PharmCAT method (as demonstrated in the following Case 5 and Case 6 examples below).

4.1) The diplotype scores are calculated for all diplotype candidates.

Diplotype score = (score of haplotype#1 - #missing positions which are the same as positions used to define the haplotype#1) + (score of haplotype#2 - #missing positions which are the same as positions used to define the haplotype#2)

4.2) The diplotype(s) with the highest score is selected as the best diplotype candidate.

<u>Examples of allele matching cases</u>

Here is an example of an allele definition table for simple demonstration. Rows represent variants and columns represent haplotypes. The first column displays the reference or ‘wild type’ haplotype (*1), while the other columns represent the ‘variants’ haplotypes. At least one of the variants of ‘variants’ haplotypes is defined by its minor allele.

variants	Haplotypes				
	*1	*2	*3	*4	*5
rs1	G	A	G	G	A
rs2	G	G	G	A	G
rs3	A	A	A	A	A
rs4	C	T	T	C	C
rs5	T	T	T	T	T

Here are demonstrations that show how to perform allele matching for different cases of sample genotypic data.

Case 1: Sample genotypes are missing at all variant positions.

Regular expression #1:
Regular expression #2:
Diplotype result : No info

Case 2: Sample genotypes are phased at all positions (with '|' symbol)

<table>
<tr><th>variants</th><th>genotypes</th></tr>
<tr><td>rs1</td><td>G|G</td></tr>
<tr><td>rs2</td><td>G|G</td></tr>
<tr><td>rs3</td><td>A|A</td></tr>
<tr><td>rs4</td><td>C|T</td></tr>
<tr><td>rs5</td><td>T|T</td></tr>
</table>

Regular expression #1: G_G_A_C_T => match with *1
Regular expression #2: G_G_A_T_T => match with *3
Diplotype result : *1/*3

Case 3: Sample genotypes are phased at some positions ('|' symbol for phased positions, '/' symbol for unphased positions)

Example 1:

<table>
<tr><th>variants</th><th>genotypes</th></tr>
<tr><td>rs1</td><td>A|G</td></tr>
<tr><td>rs2</td><td>G|A</td></tr>
<tr><td>rs3</td><td>A/A</td></tr>
<tr><td>rs4</td><td>T/C</td></tr>
<tr><td>rs5</td><td>T/T</td></tr>
</table>

Regular expression #1: A_G_(A|A)_(T|C)_(T|T) => match with *2,*5
Regular expression #2: G_A_(A|A)_(T|C)_(T|T) => match with *4

All possible combinations of haplotypes : *2/*4, *5/*4

Check all possible combinations of haplotypes that can match with sample genotypes.

<table>
<tr><td>Sample genotypes</td><td></td><td>[AG]</td><td>[GA]</td><td>[AA]</td><td>[TC]</td><td>[TT]</td></tr>
<tr><td>Diplotype *2/*4</td><td>*2</td><td>A</td><td>G</td><td>A</td><td>T</td><td>T</td></tr>
<tr><td></td><td>*4</td><td>G</td><td>A</td><td>A</td><td>C</td><td>T</td></tr>
</table>

=> *match*

Diplotype *5/*4	*5	A	G	A	C	T	
	*4	G	A	A	C	T	
					×		=> not match

Diplotype result : *2/*4

Example 2:

variants	genotypes
rs1	G|A
rs2	G|A
rs3	A/A
rs4	T/C
rs5	T/T

Regular expression #1: G_G_(A|A)_(T|C)_(T|T) => match with *1,*3
Regular expression #2: A_A_(A|A)_(T|C)_(T|T) => No match

There is "No match" in either haplotype.
Diplotype result : ?/?

Case 4: Sample genotypes are unphased at all positions (with '/' symbol) → only one regular expression is generated.

variants	genotypes
rs1	G/A
rs2	G/G
rs3	A/A
rs4	T/C
rs5	T/T

Regular expression #1: (G|A)_(G|G)_(A|A)_(T|C)_(T|T) => match with *1,*2,*3,*5
Regular expression #2: -

All possible combinations of haplotypes : *1/*1, *1/*2, *1/*3, *1/*5, *2/*2, *2/*3, *2/*5, *3/*3, *3/*5, *5/*5

Check all possible combinations of haplotypes that can match with sample genotypes.

Sample genotypes		[GA]	[GG]	[AA]	[TC]	[TT]	
Diplotype *1/*1	*1	G	G	A	C	T	
	*1	G	G	A	C	T	
		×			×		=> not match

Diplotype *1/*2	*1	G	G	A	C	T	
	*2	A	G	A	T	T	
							=> *match*
Diplotype *1/*3	*1	G	G	A	C	T	
	*3	G	G	A	T	T	
		×					=> not match
Diplotype *1/*5	*1	G	G	A	C	T	
	*5	A	G	A	C	T	
					×		=> not match
Diplotype *2/*2	*2	A	G	A	T	T	
	*2	A	G	A	T	T	
		×			×		=> not match
Diplotype *2/*3	*2	A	G	A	T	T	
	*3	G	G	A	T	T	
					×		=> not match
Diplotype *2/*5	*2	A	G	A	T	T	
	*5	A	G	A	C	T	
		×					=> not match
Diplotype *3/*3	*3	G	G	A	T	T	
	*3	G	G	A	T	T	
		×			×		=> not match
Diplotype *3/*5	*3	G	G	A	T	T	
	*5	A	G	A	C	T	
							=> *match*
Diplotype *5/*5	*5	A	G	A	C	T	
	*5	A	G	A	C	T	
		×			×		=> not match

Diplotype result : *1/*2, *3/*5

<u>Case 5</u>: From case 4, select the best candidate(s) if there is more than one diplotype candidate and the user selects the best candidate option.

From case 4:
Diplotype candidates : *1/*2, *3/*5

Identification of best diplotype candidate(s) using the scoring system (based on PharmCAT method) (https://github.com/PharmGKB/PharmCAT/wiki/NamedAlleleMatcher-101):

Each haplotype is given a score based on the number of variant positions used to define the haplotype (non-blank cells in each column). Blank cells for each column represent the same base as in *1. Reference allele (*1) will always have the maximum score because all positions are defined. If the sample data have missing positions that are required by a named haplotype definition, the position will be dropped from consideration.

variants	Haplotypes				
	*1	*2	*3	*4	*5
rs1	G	A			A
rs2	G			A	
rs3	A				
rs4	C	T	T		
rs5	T				
score	5	2	1	1	1

Diplotype score = (score of haplotype#1 - #missing positions which are the same as positions used to define the haplotype#1) + (score of haplotype#2 - #missing positions which are the same as positions used to define the haplotype#2)

Score of Diplotype *1/*2 = (5-0)+(2-0) = 7 [haplotype#1 = *1, haplotype#2 = *2]
Score of Diplotype *3/*5 = (1-0)+(1-0) = 2 [haplotype#1 = *3, haplotype#2 = *5]

The best diplotype candidate(s) are the ones with the highest score.

The best diplotype candidate(s) : *1/*2

<u>Case 6</u>: There is a missing position. Identify all diplotype candidates and select the best candidate(s) if there is more than one diplotype candidate and the user selects the best candidate option.

variants	genotypes
rs1	-
rs2	G/G
rs3	A/A
rs4	T/C
rs5	T/T

rs1 is a variant with missing data. The other variants are unphased. Only one regular expression is generated.
Regular expression #1: ._(G|G)_(A|A)_(T|C)_(T|T) => match with *1,*2,*3,*5
Regular expression #2: -

All possible combinations of haplotypes : *1/*1, *1/*2, *1/*3, *1/*5, *2/*2, *2/*3, *2/*5, *3/*3, *3/*5, *5/*5

Check all possible combinations of haplotypes that can match with sample genotypes.

Sample genotypes		[*]	[GG]	[AA]	[TC]	[TT]	
Diplotype *1/*1	*1	G	G	A	C	T	
	*1	G	G	A	C	T	
					×		=> not match
Diplotype *1/*2	*1	G	G	A	C	T	
	*2	A	G	A	T	T	
							=> *match*
Diplotype *1/*3	*1	G	G	A	C	T	
	*3	G	G	A	T	T	
							=> *match*
Diplotype *1/*5	*1	G	G	A	C	T	
	*5	A	G	A	C	T	
					×		=> not match
Diplotype *2/*2	*2	A	G	A	T	T	
	*2	A	G	A	T	T	
					×		=> not match
Diplotype *2/*3	*2	A	G	A	T	T	
	*3	G	G	A	T	T	
					×		=> not match
Diplotype *2/*5	*2	A	G	A	T	T	
	*5	A	G	A	C	T	
							=> *match*
Diplotype *3/*3	*3	G	G	A	T	T	
	*3	G	G	A	T	T	
					×		=> not match
Diplotype *3/*5	*3	G	G	A	T	T	
	*5	A	G	A	C	T	
							=> *match*
Diplotype *5/*5	*5	A	G	A	C	T	
	*5	A	G	A	C	T	
					×		=> not match

Diplotype result : *1/*2, *1/*3, *2/*5, *3/*5

Identify the haplotypes with missing positions for their defining alleles.
Missing positions: rs1
rs1 is the defining allele position of *5

Remove diplotype candidates containing *5.

Diplotype candidates now : *1/*2, *1/*3

Identification of best diplotype candidate(s)

Diplotype score = (score of haplotype#1 - #missing positions which are the same as positions used to define the haplotype#1) + (score of haplotype#2 - #missing positions which are the same as positions used to define the haplotype#2)

Diplotype *1/*2 = (5-1)+(2-1) = 5 [haplotype#1 = *1, haplotype#2 = *2]
Diplotype *1/*3 = (5-1)+(1-0) = 5 [haplotype#1 = *1, haplotype#2 = *3]

The best diplotype candidate(s) are the ones with the highest score.

The best diplotype candidate(s) : *1/*2, *1/*3
